# The Paragenital Organ of Stylopidae (Insecta: Strepsiptera) and the Functional Incorporation of the Secondary Larval Exuvia

**DOI:** 10.1002/jmor.70088

**Published:** 2025-09-25

**Authors:** Kenny Jandausch, Jakub Straka, Thomas van de Kamp, Heiko Stark, Rolf G. Beutel, Oliver Niehuis, Hans Pohl

**Affiliations:** ^1^ Institute of Zoology and Evolutionary Research Friedrich‐Schiller‐Universität Jena Jena Germany; ^2^ Department of Evolutionary Biology and Ecology University of Freiburg Freiburg Germany; ^3^ Institute for Anatomie I Jena University Hospital Jena Germany; ^4^ Department of Zoology, Faculty of Science Charles University Prague Prague Czech Republic; ^5^ Institute for Photon Science and Synchrotron Radiation (IPS) Karlsruhe Institute of Technology (KIT) Eggenstein‐Leopoldshafen Germany; ^6^ Laboratory for Applications of Synchrotron Radiation (LAS) Karlsruhe Institute of Technology (KIT) Karlsruhe Germany

**Keywords:** adaptation, cuticle thickness, endoparasitic, mating, Strepsiptera, traumatic insemination

## Abstract

Females of the insect order Strepsiptera are known to be traumatically inseminated. Traumatic insemination is the process of insemination by sperm transfer through a wound inflicted by the male in the female's integument, rather than by the male transferring sperm through the female's genital opening. Females fertilised by traumatic insemination are likely to exhibit morphological adaptations that help them to reduce the fitness costs associated with the integument wounding. One such adaptation is the presence of a paragenital organ. It has been described in traumatically inseminated bugs of the superfamily Cimicoidea and in species of the Strepsiptera genus *Stylops*. Although the paragenital organ appears to play a critical role in the mating biology of *Stylops* species, its phylogenetic roots are unknown. Here, we show that the paragenital organ in Strepsiptera may be an autapomorphy of the family Stylopidae, where we found it present in all species of the genera we studied (i.e., *Eurystylops*, *Halictoxenos*, *Hylecthrus*, *Kinzelbachus*). Our data thus refute the notion that the paragenital organ in Strepsiptera is exclusive to the genus *Stylops*. Integument relative thickness assessment based on µCT data revealed that regardless of the presence of a paragenital organ in Strepsiptera, penetration sites in the female's integument are thickened relative to control sites. In addition, we found evidence for the lateral processes of the secondary larval exuvia stabilising the paragenital organ. Our study contributes to the basic understanding of the evolution and the function of the paragenital organ in Strepsiptera and suggests potentially important morphological characters for a species‐level phylogeny of the Stylopidae.

## Graphical abstract

In the insect order Strepsiptera, we identified a paragenital organ in the family Stylopidae that serves as a secondary genital organ for traumatic insemination. We show that the cuticle at the penetration sites is thickened in all species studied, relative to control sites. Furthermore, we found that lateral projections of the secondary larval exuvia stabilise the paragenital organ, expanding its function beyond the genus *Stylops*.

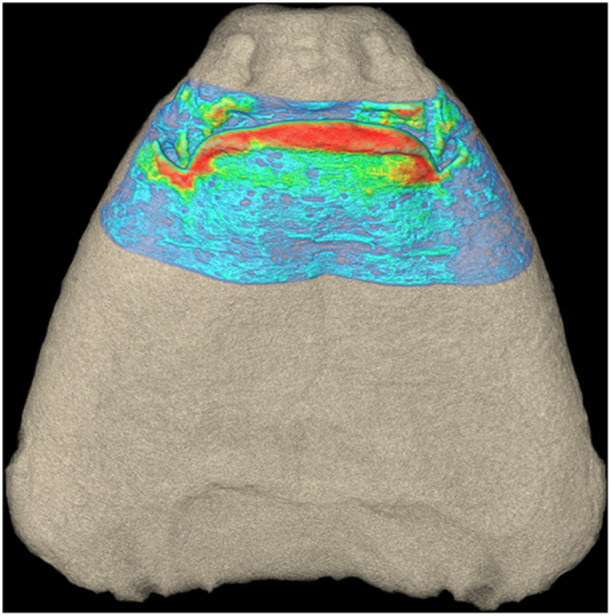

## 1 Introduction

Species of the small insect order Strepsiptera exhibit numerous derived morphological features related to an endoparasitic lifestyle. The females are known for traumatic insemination. In most species, the female is pierced by the penis close to a structure known as the birth opening. In contrast, females of the genus *Stylops* are pierced by the penis at a secondary genital structure, the paragenital organ. This cephalothoracic invagination was first described by Nassonov (1893), who hypothesised that it morphologically corresponds to an internal structure of the insect head called the tentorium and serves as a shelter for the primary larvae to retreat in emergency cases. That the structure actually functions as a secondary genital organ was discovered six decades later by Lauterbach (1954). The functional morphology of the paragenital organ has recently been studied in detail in *Stylops ovinae* (Peinert et al. 2016). However, the precise morphology of the paragenital organ, its phylogenetic distribution in Strepsiptera, and its functional interaction with the penis were hitherto unknown and are therefore the subject of the present study.

### 1.1 Strepsiptera

Strepsiptera comprise ca. 600 described species. Its species are characterised by numerous derived characters in all life stages and in both sexes (Pohl and Beutel 2005; Pohl and Beutel 2008; Straka et al. 2014; Tröger et al. 2019; Tröger et al. 2020; Benda et al. 2022; Tröger et al. 2023; Weingardt et al. 2023). All Strepsiptera species show extreme sexual dimorphism. The males are free‐living and have a short adult lifespan of a few hours. Their only function is to find females and mate with them. The females are usually obligate endoparasites in the hemocoel of other insects where they stay as secondary larvae and adults. Species of the family Mengenillidae are an exception, as their females are free‐living—a plesiomorphic character state. Species with permanently endoparasitic adult females form the monophyletic lineage Stylopidia, which comprises about 97% of all known strepsipteran species (Pohl and Beutel 2008). Female Stylopidia do not shed their larval exuvia and form a functional unit with it. This unit provides the morphological basis for the formation of a brood canal through which the primary larvae leave the maternal body cavity (Kinzelbach 1971; Pohl and Beutel 2005). Stylopidia lack a primary genital opening through which the male could transfer its sperm into the female. The same is true for Corioxenidae, the most‐early branching family within Stylopidia. Male Corioxenidae pierce the exuvia of the secondary larva with their penis, usually in the region of the female's mouth or on a membranous pleural area of the prothoracic region (Kirkpatrick 1937; Pohl and Beutel 2008). Females of the lineage Stylopiformia (Stylopidia excl. Corioxenidae) are oriented with their ventral side facing away from the host. They have a secondary birth opening that connects the birth organs to the external environment via the brood canal. The secondary orifice also serves as an exit for the primary larvae from the female's body. However, in species of Stylopiformia, it usually serves as the exclusive site where the penis penetrates the female's cuticle during traumatic insemination (Grabert 1953; Lauterbach 1954; Peinert et al. 2016; Jandausch et al. 2022; Jandausch et al. 2023). Species of the genus *Stylops* are a known exception, as they possess a paragenital organ where penetration occurs instead.

### 1.2 The Paragenital Organ

Lauterbach (1954) discovered the paragenital organ, which he named ‘Brutkanaltasche.’ He described it as a pocket‐shaped invagination of the integument at the anterior end of the brood canal and identified it as the exclusive site of penetration and sperm transfer in *S. ovinae* (= *Stylops ater* sensu Straka et al. (2015)). Consequently, he considered the paragenital organ as functionally equivalent to a bursa copulatrix. Löwe and Beutel (2016) also concluded that the paragenital organ of *S. ovinae* represents a cephalothoracic invagination and reported that the ventral wall of the invagination cuticle is thickened and rests on a multilayered epidermis. The authors found the same features (i.e., thickened cuticle, multilayered epidermis) in species lacking a paragenital organ such as *Elenchus tenuicornis* (Elenchidae), *Tridacylophagus etoi* (Halictophagidae), and *Xenos vesparum* (Xenidae) (Peinert et al. 2016; Richter et al. 2017; Jandausch et al. 2022; Jandausch et al. 2023). However, whether this paragenital organ is unique to the genus *Stylops* or also occurs in other lineages of Stylopiformia has not been investigated yet.

Even though the paragenital organ was first described more than 70 years ago, morphological studies of this unique structure have been largely limited to reporting its presence or absence. Analyses of its specific morphological features and more detailed structural investigations were almost entirely lacking. An intriguing example are the processes lateral to the paragenital organ in the second larval exuvia, which Kinzelbach (1971) described as a tentorium. While it was noted that these processes extend into the lateral parts of the paragenital organ, they have never been addressed in any subsequent study. As this character complex is potentially phylogenetically informative and likely played a role in the evolution of the group, our aim was to provide a more detailed description of the paragenital organ and its associated structures.

### 1.3 Copulatory Interaction: Paragenital Organ and Aspects of Penis Morphology

In their work on the copulatory behaviour of *S. ovinae*, Peinert et al. (2016) highlighted the potential importance of the paragenital organ in the process of traumatic insemination. Specifically, the authors suggested that the structural properties of the paragenital organ could reduce trauma caused by multiple copulations. Thus, the paragenital organ could be considered functionally analogous to the spermalege of bed bugs (Reinhardt et al. 2003; Michels et al. 2015). The interpretation of the paragenital organ as an adaptation reducing inflicted wounds in traumatically inseminated females was further supported by recent experiments analysing penetration forces and structural composition at penetration and control sites in *S. ovinae* and *X. vesparum* (Jandausch et al. 2022). The authors found that the cuticle of both species was consistently thickened at penetration sites compared to control sites. This feature likely supports wound closure and thus reduces the negative effects of traumatic insemination. The authors also reported the occurrence of a paragenital organ in two other *Stylops* species: *S. hammella* and *S. melittae*. This structural complex may also serve as a prezygotic mating barrier preventing heterospecific mating. Using behavioural experiments and morphological covariation analyses, Jandausch et al. (2022) found that *S. ovinae* females attract males of several sympatric *Stylops* species, but that only conspecific males can successfully mate. It thus appears likely that the paragenital organ and the penis act as a heterospecific mating barrier in this genus. In particular, the size of the penis and the acumen—the sharp tip of the penis (Kinzelbach 1971)—seem plausible elements of a lock‐and‐key mechanism, as found in various groups of arthropods (Jandausch et al. 2022). Therefore, one aim of our study was to assess possible correlations between features of the penis and the corresponding paragenital organs.

### 1.4 Aims of the Present Study

In this study, we (1) present for the first time a detailed description of the paragenital organ, defining several specific features with potential phylogenetic relevance based on detailed µCT‐based 3D reconstructions of the female cephalothorax; (2) emphasise the exuvial lateral processes, which apparently interact with the paragenital organ, and discuss their potential function; (3) investigate the phylogenetic distribution of the paragenital organ in Stylopidae, using Corioxenidae and Xenidae as outgroups; (4) discuss the function and role of the paragenital organ in copulation, particularly considering the thickness of the integument at penetration sites; and (5) discuss possible interactions of the paragenital organ and the penis with respect to potentially correlated structural features.

## 2 Materials and Methods

### 2.1 Microcomputed Tomography and 3D Reconstruction

With the exception of females of *Stylops melittae* and *Triozocera macroscyti* (Table 1), all specimens were scanned in pure ethanol at the Imaging Cluster of the Karlsruhe Institute of Technology Synchrotron Radiation Facility, using a polychromatic X‐ray beam generated by a 1.5 T bending magnet and spectrally filtered by 0.5 mm Al. A fast indirect detector system consisting of a 13 µm LSO:Tb scintillator (Cecilia et al. 2011), a diffraction‐limited optical microscope (Optique Peter) (Douissard et al. 2012), and a 12‐bit pco. dimax high‐speed camera with 2016 × 2016 pixels was used. Scans were derived from 3,000 projections (each 0.06°) at 70 fps (exposure time of 142 ms) over an angular range of 180°. An optical magnification of 10x resulted in an effective pixel size of 1.22 µm. The concert control system was used for automated data acquisition, and online reconstruction of tomographic slices was used for data quality assurance (Vogelgesang et al. 2016). Data processing included flat field correction and phase retrieval of the projections based on the intensity transport equation (Paganin et al. 2002). The X‐ray beam parameters for the algorithms in the data processing pipeline were calculated using *syris* (Faragó et al. 2017). The execution of the pipelines, including tomographic reconstruction, was performed with the UFO framework (Vogelgesang et al. 2012).

**Table 1:** Sex, species, family, locality, date, and collectors of specimens analysed.

Species	Family	Locality	Date	Collector	Identifier
Female specimens					
*Triozocera macroscyti* Esaki & Hashimoto, 1958	Corioxenidae	Japan, Nagano campus area Shinshu University Faculty of Agriculture	05/2017	Y. Nakase	Y. Nakase
*Paraxenos erberi* Saunders, 1872	Xenidae	Slovakia, Virt env., sand dune pasture	06/2018	J. Straka	J. Straka
*Deltoxenos lusitanicus* (Luna de Carvalho, 1960)	Xenidae	Czechia, Hradec Králové, PP Na Plachtě	08/2007	P. Bogusch	D. Benda
*Tuberoxenos sphecidarum* (Siebold, 1839)	Xenidae	Mongolia, Arvaykheer, 137 km NE, Övörkhangay prov.	07/2004	J. Straka	J. Straka
*Xenos vesparum* (Rossi, 1793)	Xenidae	Breeding ex. *Polistes dominula* (Germany, Thüringen, Rothenstein) infected with primary larva, DE Baden‐Württemberg, Kaiserstuhl Oberbergen	06/2019	H. Pohl, F. Schweitzer	H. Pohl, F. Schweitzer
*Crawfordia warnckei* Kinzelbach, 1970	Stylopidae	Morocco, Idelsane env., Ouarzazate prov.	03/2019	D. Benda	D. Benda
*Eurystylops ogloblini* Benda & Straka, 2025	Stylopidae	Türkiye, Budaklı env., Kahramanmaraş prov.	07/2011	J. Straka	—
*Halictoxenos arnoldi* Perkins, 1918	Stylopidae	Türkyie, Budaklı env., Kahramanmaraş prov.	07/2011	J. Straka	J. Straka
*Halictoxenos simplices* Noskiewicz & Poluszyński, 1924	Stylopidae	Hungary, Dunatetétlen env., salt marshes	06/2013	D. Benda, P. Bogusch, J. Straka	J. Straka
*Hylecthrus rubi* Saunders, 1850	Stylopidae	Hungary, Bikolpuszta, loess steppe	08/2018	D. Benda	D. Benda
*Kinzelbachus friesei* (Hofeneder, 1949)	Stylopidae	Tajikistan, Firdavsi/Buragen env., 6 km SW Shakriston	07/2019	J. Straka	J. Straka
*Stylops hammella* Perkins, 1918	Stylopidae	Czechia, Havraníky, Znojmo env.	05/2017	J. Straka	J. Straka
*Stylops sapmiensis* Lähteenaro & Bergsten, 2025	Stylopidae	Poland, Gozdnica, sandpit	04/2015	K. Kodejš, J. Straka	J. Straka
*Stylops melittae* Kirby, 1802	Stylopidae	Czechia, Stroupeč env., PP Stroupeč	04/2016	P. Bogusch	P. Bogusch
*Stylops nassonowi* Pierce, 1909	Stylopidae	Czechia, Pavlov env., NPR Děvín‐Kotel‐Soutěska	04/2016	P. Bogusch	P. Bogusch
*Stylops ovinae* Noskiewicz & Poluszyński, 1928	Stylopidae	Czechia, Káraný nad Labem, V Dejmlovce	03/2008	J. Batelka	J. Straka
*Stylops spretus* Perkins, 1918	Stylopidae	Czechia, Káraný nad Labem, V Dejmlovce	03/2008	J. Batelka	J. Straka
Male specimens					
*Tuberoxenos sphecidarum* (Siebold, 1839)	Xenidae	Mongolia, Arvaykheer, 137 km NE, Övörkhangay prov.	07/2004	J. Straka	J. Straka
*Xenos vesparum* (Rossi, 1793)	Xenidae	Germany, Rheinland‐Pfalz, Mettenheim	07/2020	H. Pohl, K. Jandausch	H. Pohl, K. Jandausch
*Stylops hammella* Perkins, 1918	Stylopidae	Czechia, Dolní Věstonice, Pálava	04/2014	J. Straka	J. Straka
*Stylops sapmiensis* Lähteenaro & Bergsten, 2025	Stylopidae	Poland, Gozdnica, sandpit	04/2015	K. Kodejš, J. Straka	J. Straka
*Stylops melittae* Kirby, 1802	Stylopidae	Czechia, Písečný vrch, České středohoří	02/2008	J. Batelka	J. Straka
*Stylops nassonowi* Pierce, 1909	Stylopidae	Czechia, Dymokury env., pond bank	05/2012	J. Straka	J. Straka
*Stylops ovinae* (*S. ater* sensu Straka et al. (2015))	Stylopidae	Czechia, Hlásná Třebáň, štěrková cesta u zahr. Kol., Český Kras	03/2011	P. Špryňar	J. Straka
*Stylops spretus* Perkins, 1918	Stylopidae	Czechia, Kamýk, Praha‐Modřany	04/2013	J. Straka	J. Straka

*Note:* All specimens were preserved in absolute Ethanol.

To improve the contrast in some µCT scans, the female of *Stylops mellitae* was stained in an iodine solution (1% iodine in 100% ethanol) for 3 days. It was then transferred to pure ethanol and scanned in a SkyScan221 µCT (Max Planck Institute for the History of Mankind, Jena, Germany) with a beam strength of 40 kV and 300 mA. Images were taken every 0.2° in a 360° scan, with an exposure time of 5800 ms. The resulting final pixel size was 1.22 µm.

The female of *Triozocera macroscyti* was dried at the critical point using an Emitech K850 Critical Point Dryer with liquid CO_2_ (Sample Preparation Division, Quorum Technologies, Ashford, England). The sample was scanned in a SkyScan221 µCT (Max Planck Institute for the History of Mankind, Jena, Germany) with a beam strength of 70 kV and 300 mA. Images were taken every 0.15° in a 360° scan, with an exposure time of 3,000 ms. The resulting final pixel size was 0.6 µm.

### 2.2 Segmentation of Cuticle

We pre‐segmented tomographic data using Amira 6.0.1 and completed the segmentation semi‐automatically using the online platform Biomedisa (Lösel et al. 2020). The segmented material of the cuticle was then exported from Amira as a TIFF‐file stack using the ‘multiExport’ plugin script (Engelkes et al. 2018) for visualisation and rendering in VG Studiomax 2.0.5 (Volume Graphics, Heidelberg, Germany). In addition, the segmented material was exported as a binary NIFTI‐file, and the mesh created with the marching cubes algorithm as an OBJ file for the subsequent relative cuticle thickness assessment.

### 2.3 Relative Cuticle Thickness Assessment

Cuticle thickness was assessed quantitatively, but only the relative thickness within each species. We consider comparisons of absolute thickness values across different species as unreliable due to variation in body size. To avoid this source of potential misinterpretation, we present no absolute values in our study. The relative thickness differences were visualised using a colour gradient from thin to thick.

Relative cuticle thickness assessments were performed using the ‘scalar measure. thickness’‐function of ImageXd 6.2.6 (Heiko Stark, Jena, Germany, URL: https://starkrats.de). We assessed the relative cuticle thickness at the penetration sites and in the surrounding regions of the integument based on the segmented cuticles achieved from the µCT‐scans. Relative cuticle thickness assessments were made (1) using voxel‐based data (NIFTI) and (2) using mesh data (OBJ) from 3D modelling.

(1) In the first approach, the spatial environment around each voxel was radially scanned for similar grayscale values within a 90‐voxel radius. The ray with the highest count of similar grayscale voxels was then stored as the maximum value at the corresponding position in a second data set. This second data set thus represents the encoded thickness at the exact location of the integument. It then served as a template for the colour visualisation of local thickness. For this purpose, the local values were coloured and visualised using a predefined colour palette in VG Studiomax. Points with high voxel counts were encoded in red (thick) according to the chosen gradient, while low voxel counts were encoded in blue (thin). In VG Studiomax, the data were rendered for publication.

(2) In the second approach, the thickness was not measured radially from a single voxel. Instead, measurements were performed from the vertex points of a mesh along the inverse normal vector of a given structure. The voxel count measured within a 105‐voxel radius was then directly colour‐coded according to the previously described colour scheme and stored at the respective vertex point. The resulting mesh (3D model) was subsequently visualised using Blender 2.82.7 (Blender Foundation, Amsterdam, The Netherlands). Models were slightly smoothed, and the vertex count was reduced by 30%. To complement the 2D renders presented in this publication, all models of the relative cuticle measurements were uploaded to Sketchfab using the Sketchfab Blender Addon 1.5.0 (The Khronos Group Beaverton, USA). This allows readers to access the results in a three‐dimensional, interactive format.

### 2.4 Image Processing

Adobe Photoshop 21.2.1 (Adobe Systems, San Jose, USA) was used to process images and arrange them into plates. Adobe Illustrator 24.2.1 (Adobe Systems, San Jose, USA) was used for labelling plates and drawings.

### 2.5 Specimens

In the description of the penis, we followed the terminology of Kinzelbach (1971).

## 3 Results

### 3.1 **Description of the Paragenital Organ** (Figure 1)

**Figure 1:**
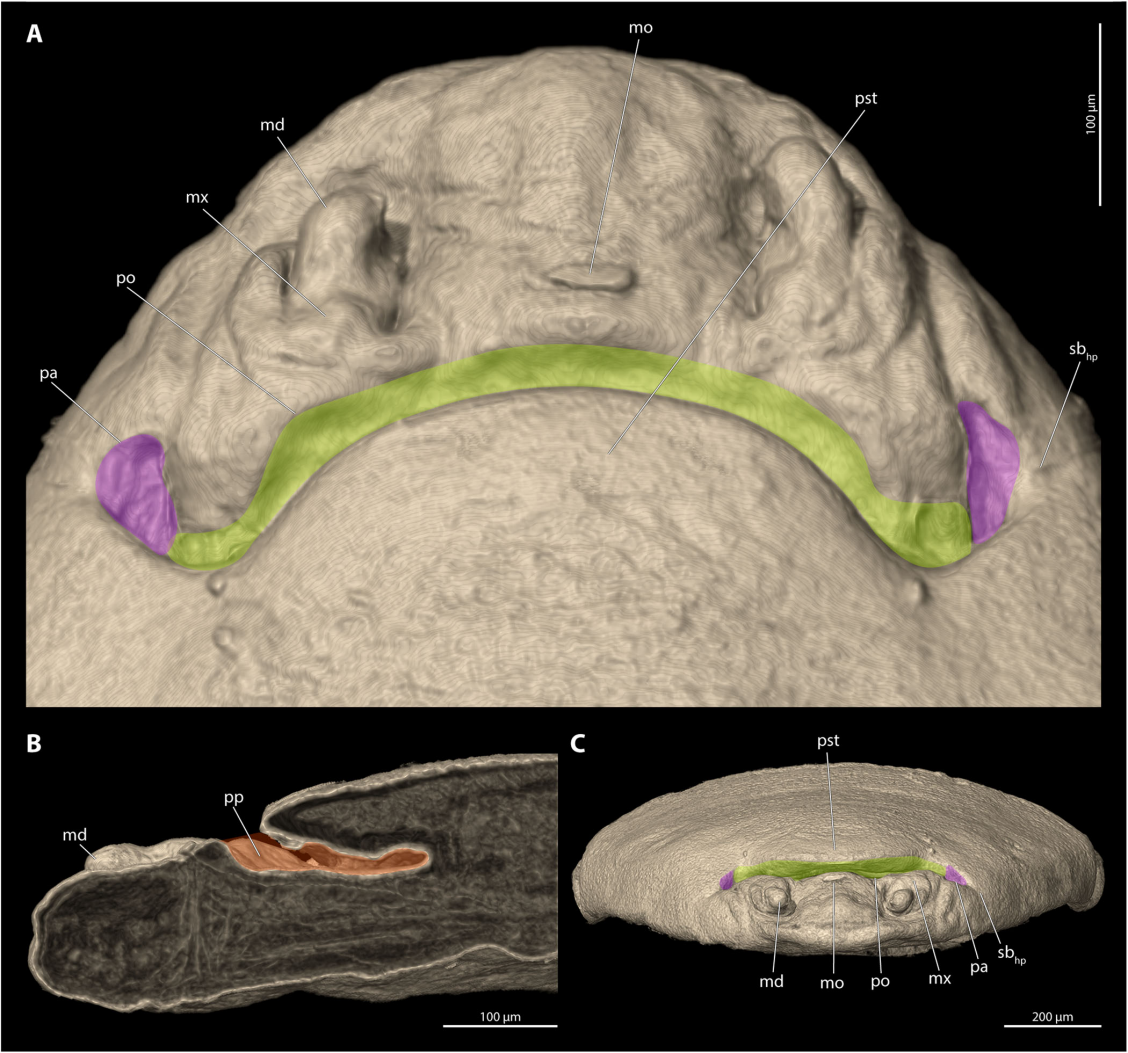
Cephalothorax (larval exuvia removed) of *Stylops mellittae* illustrating the general morphology of the paragenital organ. (A) Ventral view. (B) Mediosagittal section. (C) Frontal view. Shown are the opening of the paragenital organ (green), the paragenital auricles (pink), and the paragenital pouch (orange). md, mandible; mo, mouth opening; mx, maxilla; pa, paragenital auricle; po, paragenital opening; pp, paragenital pouch; pst, prosternum; sb_hp_, segmental boundary between head and prothorax.

**Figure 2:**
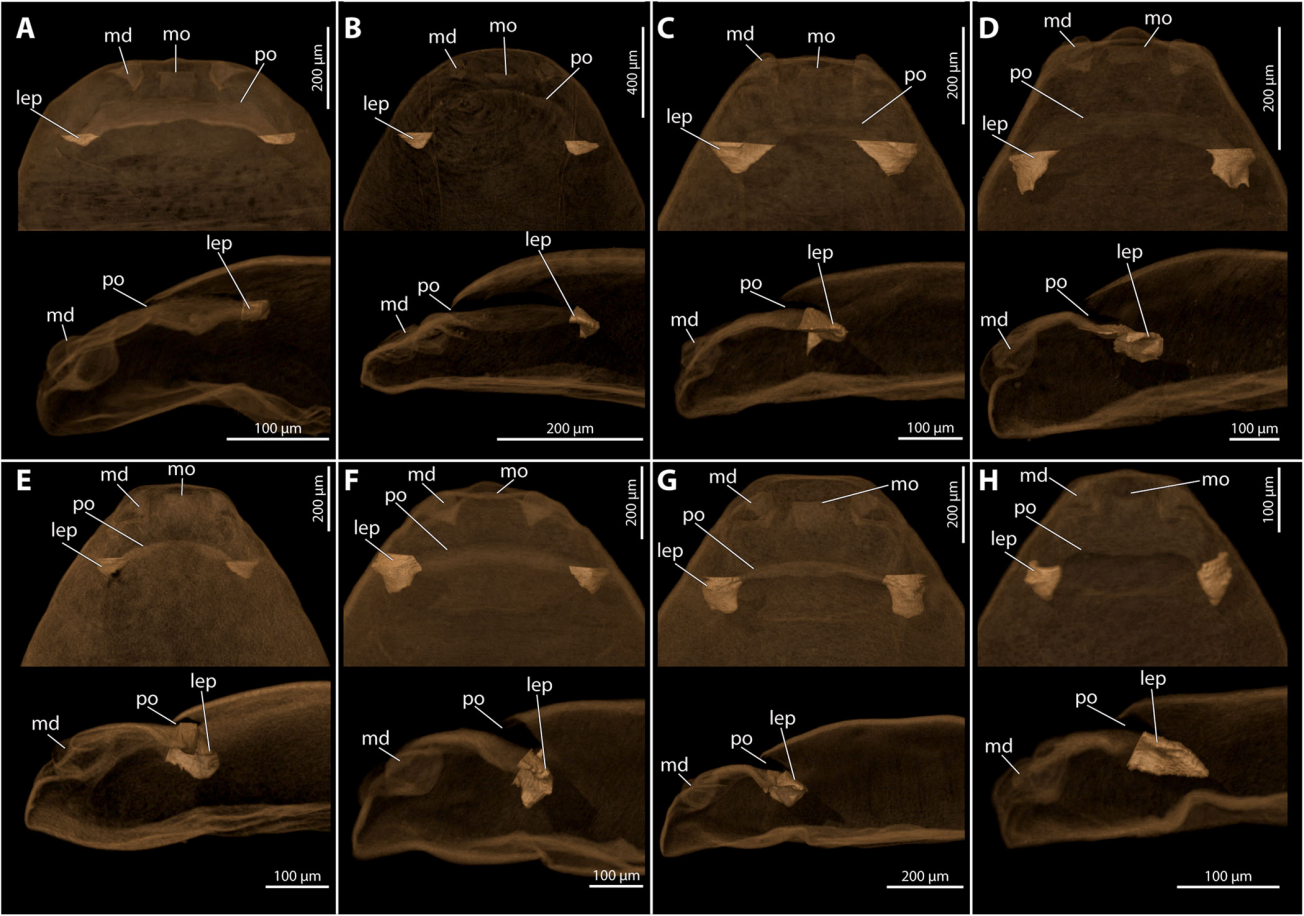
Anterior parts of the female cephalothorax with the internal cuticular processes highlighted. (A) *Eurystylops ogloblini* (B) *Kinzelbachus friesei* (C) *Stylops hammella* (D) *Stylops sapmiensis* (E) *Stylops melittea* (F) *Stylops nassonowi* (G) *Stylops ovinae* (H) *Stylops spretus*. The top row shows ventral view; the bottom row depicts mediosagittal sections. ep, lateral exuvial process; md, mandible; mo, mouth opening; po, paragenital opening.

The paragenital organ is an invagination of the integument of the adult female, located on the ventral side between the head and the prothoracic region. Its opening is transverse and fissure‐like, located on the ventral side, extending almost from one side to the other in most examined species. Membranous folds are present at each lateral end of the opening (hereafter referred to as paragenital auricles), in several species with strongly sclerotised cuticular projections of the exuvia of the secondary larva extending into their lumen (Figure 2). These lateral exuvial projections are absent in *Halictoxenos*, weakly developed in *Kinzelbachus* and *Eurystylops*, and distinct in *Stylops* (Figure 2). The paragenital opening is externally covered by a thin membrane of the secondary larval exuvia (e.g., approximately 2 µm thick in *S. ovinae;* (Löwe and Beutel 2016; Peinert et al. 2016; Richter et al. 2017). We assume that this membrane is ruptured when the penis enters the paragenital organ during copulation. The paragenital organ extends into the body lumen as a shallow pouch, reaching different depths in different species. In the case of deep invaginations (found in species of *Stylops*), the pouch extends obliquely towards the dorsal side of the female body and ends in the prothoracic region (e.g., Figures 9 and 10). The cuticle of the ventral wall of the paragenital organ is always significantly thickened compared to the dorsal wall and to other cuticular areas, such as for instance the anterior region of the brood canal. The processes of the secondary larval exuvia extend into the paragenital pouch to varying degrees on each side of the paragenital opening.

### 3.2 Detailed Description of the Paragenital Organ

The detailed description of each species in terms of paragenital organ and integument thickness is followed by a summarising table (Table 2). 3D models are available on sketchfab. Links are provided in Table 3.

**Table 2:** Summary of the results.

Family	Species	Brood canal opening	Paragenital organ	Shape of paragenital opening	Paragenital auricles	Paragenital pouch	Lateral exuvial process	Thickened area of the Integument	Figures
**Corioxenidae**	*Triozocera macroscyti*	absent	absent	absent	absent	absent	absent	thin area around the mouth	3
**Xenidae**	*Paraxenos erberi*	present	absent	absent	absent	absent	absent	—	4
	*Tuberoxenos sphecidarum*	present	absent	absent	absent	absent	absent	—	4
	*Deltoxenos lusitanicus*	present	absent	absent	absent	absent	absent	—	4
	*Xenos vesparum*	present	absent	absent	absent	absent	absent	anterior parts of the brood canal	5
**Stylopidae**	*Crawfordia warnckei*	present	absent	absent	absent	absent	absent	—	6
	*Eurystylops ogloblini*	present	present	sinuate in ventral view, with a flat and elongated apex	absent	weakly pronounced, (12.9% of the cephalothorax length)	present	ventral wall of paragenital organ	2, 6
	*Halictoxenos arnoldi*	present	present	flattened arch in ventral view	absent	weakly pronounced, (3.3% of the cephalothorax length)	absent	—	7
	*Halictoxenos simplicis*	present	present	arcuate and very slit	absent	weakly pronounced, (11.4% of the cephalothorax length)	absent	ventral wall of paragenital organ, with a massive lobe	7
	*Hylecthrus rubi*	present	present	very slightly curved and is bent posterad	absent	reaches deep into the body cavity, (25% of the cephalothorax length)	absent	ventral wall of paragenital organ, with a massive lobe	8
	*Kinzelbachus friesei*	present	present	strongly arched, with a flat apex	weakly developed	does not reach deep into the body cavity, (6.3% of the cephalothorax length)	present	very slightly thickened	2, 8
	*Stylops hammella*	present	present	slightly arched with a flattened apex	weakly developed	reaches deep into the body cavity, (20.6% of the cephalothorax length)	present	ventral and dorsal walls of the paragenital organ	2, 9
	*Stylops sapmiensis*	present	present	arched with a flattened apex	well developed	reaches deep into the body cavity, (16.7% of the cephalothorax length)	present	ventral wall of the paragenital organ	2, 9
	*Stylops melittae*	present	present	curved	well developed	reaches deep into the body cavity, (21% of the cephalothorax length)	present	ventral wall of the paragenital organ	2, 10
	*Stylops nassonowi*	present	present	almost straight with only a slight curvature	well developed	reaches deep into the body cavity, (20% of the cephalothorax length)	present	ventral wall of the paragenital organ	2, 10
	*Stylops ovinae*	present	present	almost straight with only a slight curvature	well developed	reaches deep into the body cavity, (20.7% of the cephalothorax length)	present	ventral wall of the paragenital organ	2, 11
	*Stylops spretus*	present	present	slightly concave at the apex and trapezoidal in appearance	does not reach deep into the body cavity	does not reach deep into the body cavity, (12.1% of the cephalothorax length)	present	ventral wall of the paragenital organ	2, 11

Abbreviation: “—” indicates missing information.

**Table 3:** Links to interactive models of female cephalothoraces on Sketchfab.

Family	Species	Link
**Corioxenidae**	*Triozocera macroscyti*	https://sketchfab.com/3d-models/triozocera-macroscyti-013527febb31487882edb91a58edc875
**Xenidae**	*Paraxenos erberi*	https://sketchfab.com/3d-models/paraxenos-erberi-f1fd46c0c2504d048bdc4738013c6f11
	*Tuberoxenos sphecidarum*	https://sketchfab.com/3d-models/tuberoxenos-sphecidarum-e1fd36a1b3724da7a077dd9526cf74c6
	*Deltoxenos lusitanicus*	https://sketchfab.com/3d-models/deltoxenos-lusitanicus-c1ea4259ccfb4edc821d5a9f9e23ebf5
	*Xenos vesparum*	https://sketchfab.com/3d-models/xenos-vesparum-a0854c786aad4f26be3a7e7080765bb1
**Stylopidae**	*Crawfordia warnckei*	https://sketchfab.com/3d-models/crawfordia-warnckei-bc369e3d57b24b3a87c769dd77e6e707
	*Eurystylops ogloblini*	https://sketchfab.com/3d-models/eurystylops-ogloblini-be61ab9276b44cd89345e642de4ec482
	*Halictoxenos arnoldi*	https://sketchfab.com/3d-models/halcitoxenos-arnoldi-1bd2cdb41c9c4a29b0126fafc3b49338
	*Halictoxenos simplicis*	https://sketchfab.com/3d-models/halictoxenos-simplicis-7197e049487e49d29b65a0a57d43da41
	*Hylecthrus rubi*	https://sketchfab.com/3d-models/hylechtrus-rubi-d4e1b2efb3a64a97b965d643e6a7a295
	*Kinzelbachus friesei*	https://sketchfab.com/3d-models/kinzebachus-friesei-62b749e96d6b4d52acd3e357541ffa38
	*Stylops hammella*	https://sketchfab.com/3d-models/stylops-hammella-5f2939e6cb534dcc8a07c8794e900216
	*Stylops sapmiensis*	https://sketchfab.com/3d-models/stylops-sapmiensis-8ca943b9e20f493b99387be60cbe9822
	*Stylops melittae*	https://sketchfab.com/3d-models/stylops-melittae-88c6931fbeb840f4843a4f17fd4f33b2
	*Stylops nassonowi*	https://sketchfab.com/3d-models/stylops-nassonowi-9d48998f69e34fd8824ffaaf39c959e3
	*Stylops ovinae*	https://sketchfab.com/3d-models/stylops-ovinae-be230487bb484f2895d721e39d538716
	*Stylops spretus*	https://sketchfab.com/3d-models/stylops-spretus-35b8a96e1f6940009a6edbe0f8e8ba97

### 3.3 Corioxenidae

#### 3.3.1 *Triozocera macroscyti* (Figure 3)

**Figure 3:**
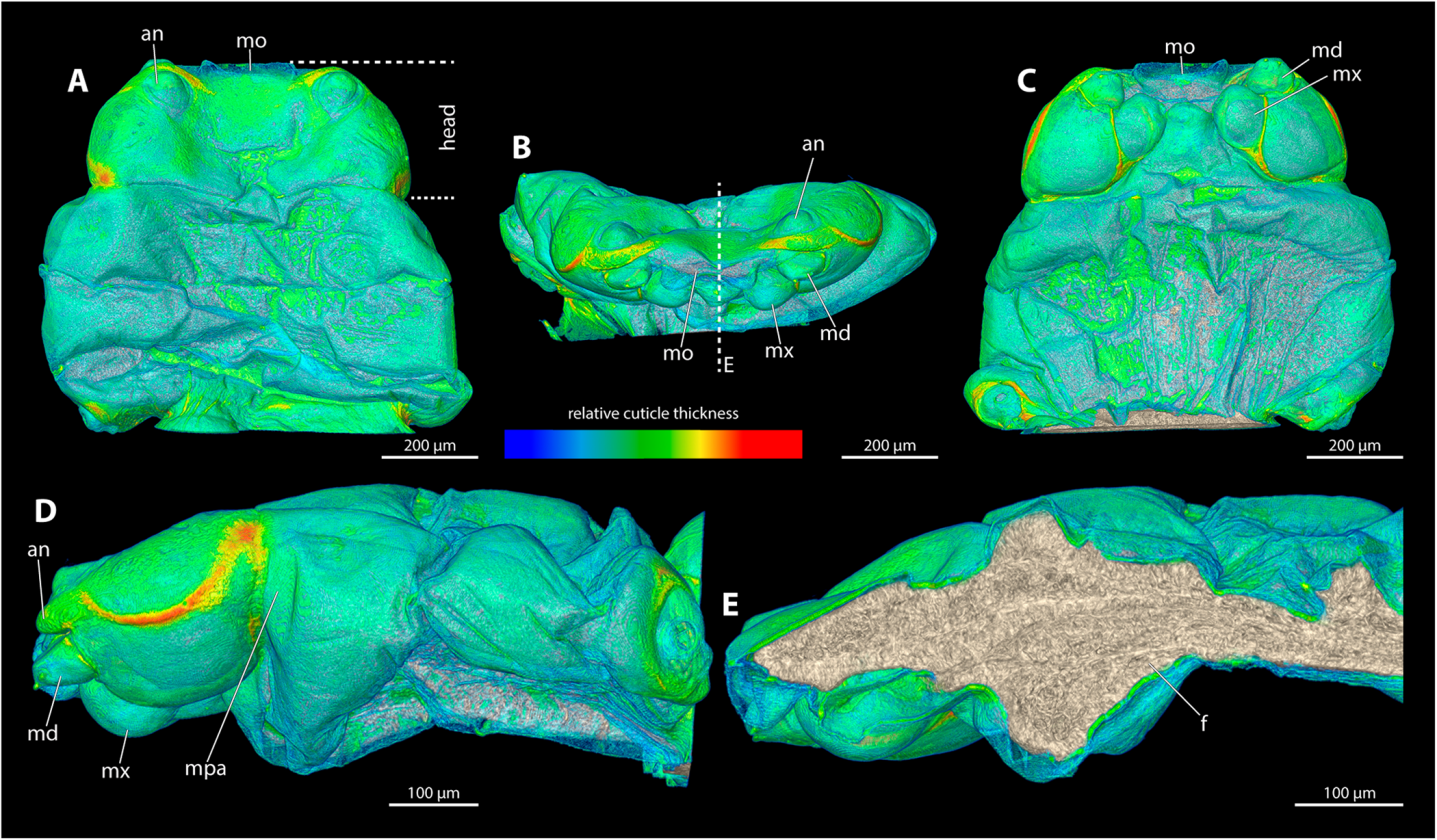
Relative thickness of the cephalothorax cuticle of *Triocozera macroscyti*. (A) Exuvia of female secondary larva (dorsal view). (B) Exuvia of female secondary larva (frontal view). (C) Adult female cephalothorax with exuvia of secondary larva (ventral view). (D) Female cephalothorax with exuvia of secondary larva (lateral view). (E) Female cephalothorax (beige) within exuvia of secondary larva in colour correlated with thickness of the exuvia (mediosagittal section). Blue colour indicates thin cuticle, red thickened cuticle. an, antennal bud; f, female; md, mandible; mo, mouth opening; mpa, membranous pleural area; mx, maxilla.

The paragenital organ is absent in *T. macroscyti*. The exuvia of the secondary larvae lacks a brood canal opening. Since penetration in Corioxenidae occurs by piercing the exuvia of the female secondary larva, we examined the relative thickness of this exuvia. We identified a weakly sclerotised area in the oral region and found that the integument in membranous pleural areas was not thickened compared to surrounding regions.

### 3.4 Xenidae

#### 3.4.1 *Paraxenos erberi* (Figure 4A–F)

**Figure 4:**
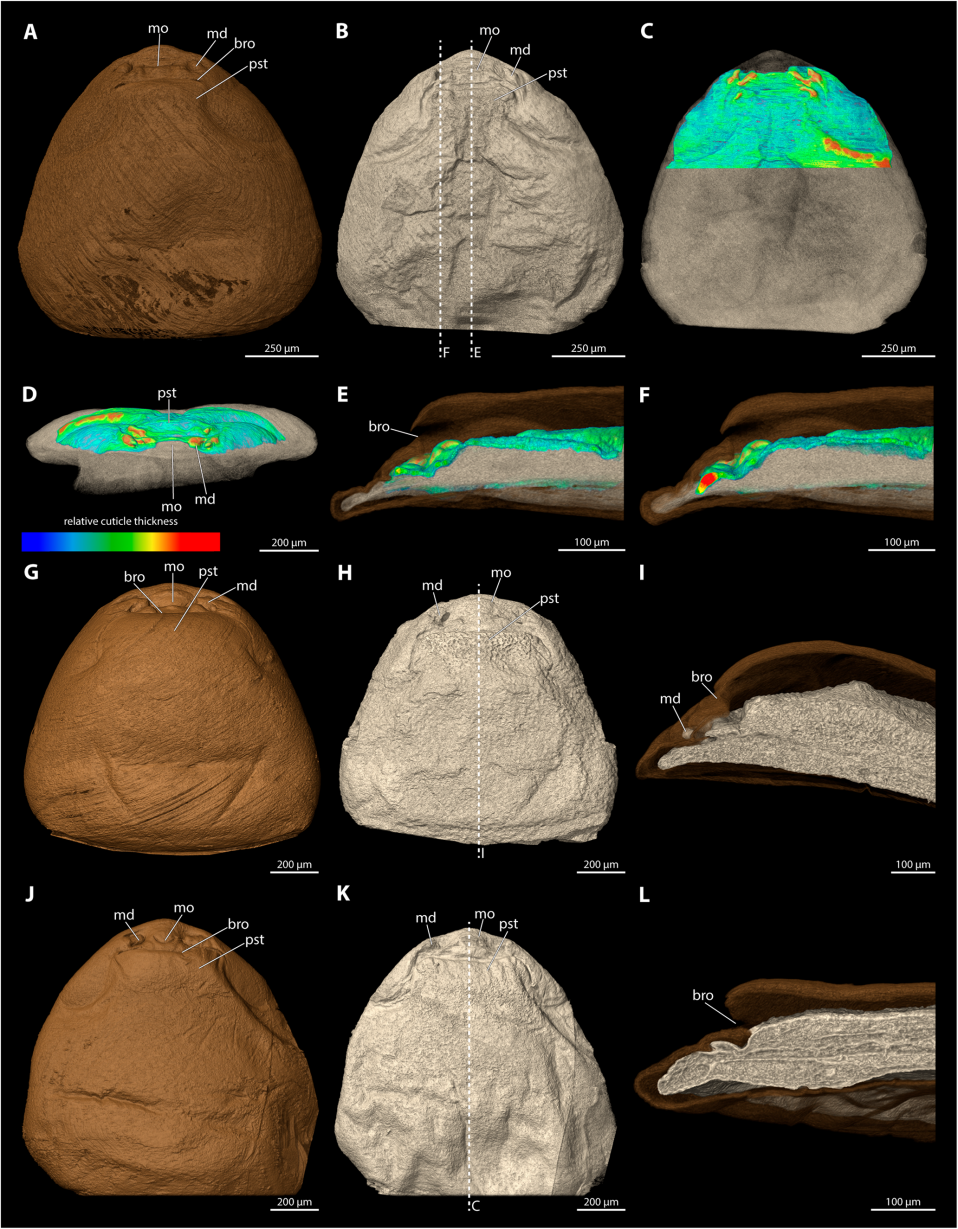
Female cephalothoraces of *Paraxenos erberi* (A–F), *Tuberoxenos sphecidarum* (G–I), and *Deltoxenos lusitanicus* (J–L), with the ventral side facing upwards. Relative thickness of the cuticle indicated by colour. Blue areas represent thin cuticular regions, while red areas represent thickened areas. The relative cuticle thickness of *T. sphecidarum* and *D. lusitanicus* could not be assessed with the available µCT data. (A, G, J) Exuvia of the secondary larva. (B, H, K) Female cephalothorax, exuvia of secondary larva removed. (C) Female cephalothorax (D) Frontal view. (E) Mediosagittal section. (F) Sagittal view. (I, J) Female cephalothorax (mediosagittal section). bro, birth opening; md, mandible; mo, mouth opening; pst, prosternum.

A paragenital organ is absent. We did not observe any thickening of the female cuticle along the anterior portions of the brood canal, where penetration occurs. Thickened integument is only present in the region of the mouthparts and their articulations.

#### 3.4.2 *Tuberoxenos sphecidarum* (Figure 4G–I)

A paragenital organ is absent. Relative cuticular thickness could not be assessed with the available µCT data.

#### 3.4.3 *Deltoxenos lusitanicus* (Figure 4J–L)

*D. lusitanicus* lacks a paragenital organ. It was not possible to assess the relative thickness of the cuticle with the available µCT data.

#### 3.4.4 *Xenos vesparum* (Figure 5)

**Figure 5:**
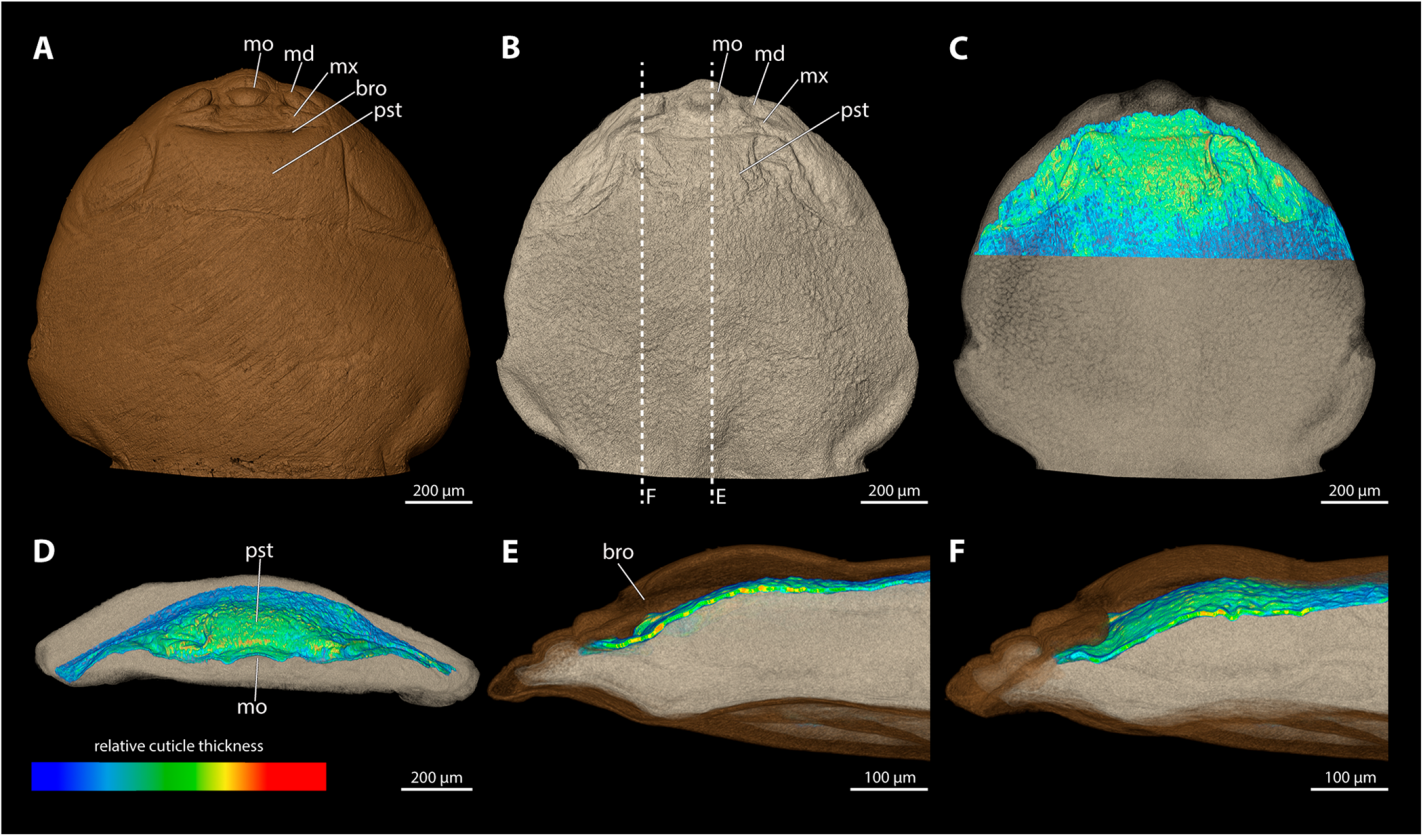
Female cephalothorax of *Xenos vesparum*, with the ventral side facing upwards. Relative thickness of the cuticle indicated by colour. Blue areas represent thin cuticular regions, while red areas represent thickened areas. (A) Exuvia of the secondary larva. (B) Female cephalothorax, exuvia of secondary larva removed. (C) Female cephalothorax (D) Frontal view. (E) Mediosagittal section. (F) Sagittal view. bro, birth opening; md, mandible; mo, mouth opening; mx, maxilla; pst, prosternum.

*X. vesparum* lacks a paragenital organ. A thickened cuticle was observed in the anterior regions of the brood canal. The thickness of the cuticle decreases posterad, until it reaches the metathoracic region.

### 3.5 Stylopidae

#### 3.5.1 *Crawfordia warnckei* (Figure 6A–C)

**Figure 6:**
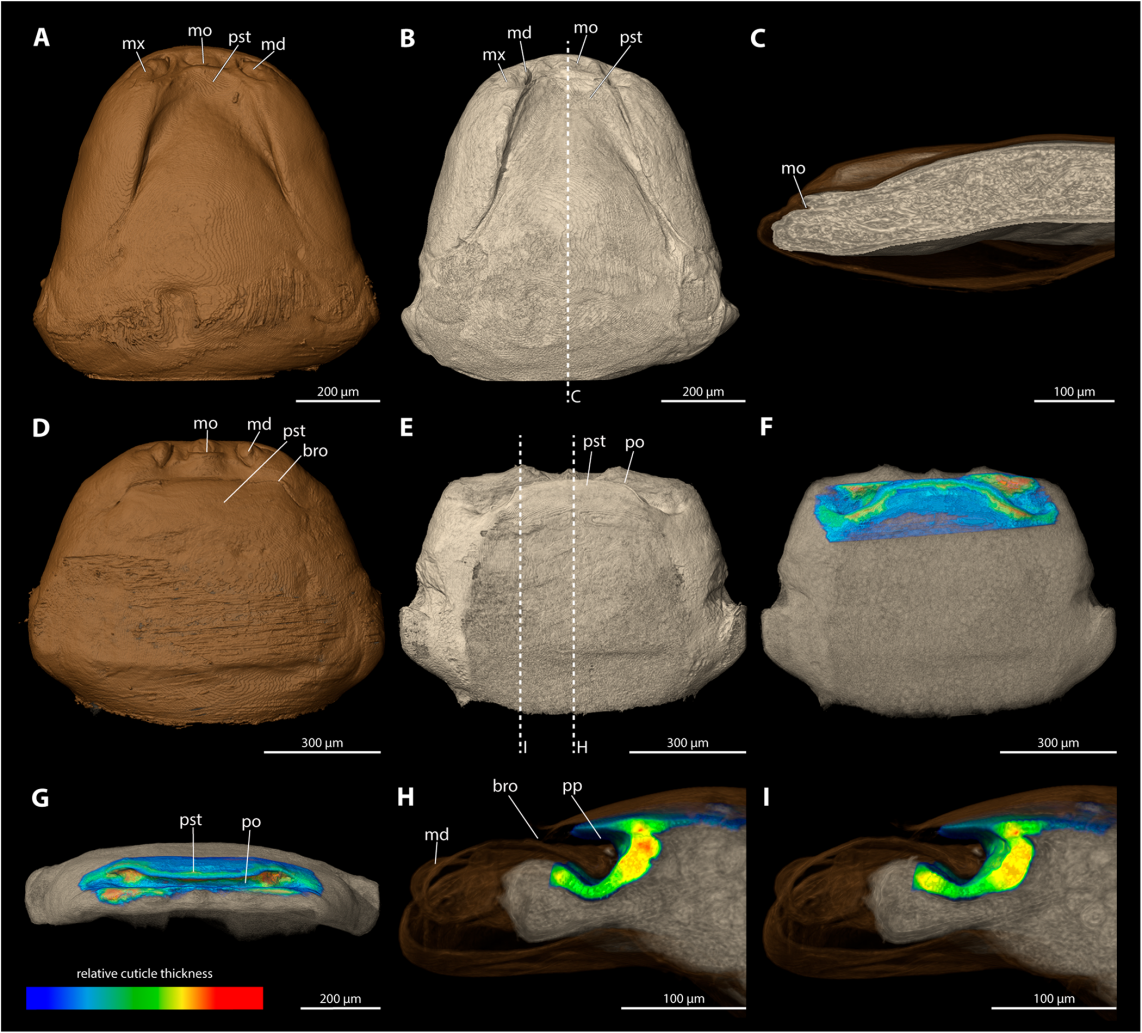
Female cephalothoraces of *Crawfordia warnckei* (A–C) and *Eurystylops ogloblini* (D–I) with the ventral side facing upwards. Relative thickness of the cuticle indicated by colour. Blue areas represent thin cuticular regions, while red areas represent thickened areas. The available µCT data did not allow to assess the relative cuticle thickness of *C. warnckei*. (A, D) Exuvia of the secondary larva. (B, E) Female cephalothorax, exuvia of secondary larva removed. (C) Female cephalothorax (mediosagittal section). (F) Female cephalothorax. (G) Frontal view. (H) Mediosagittal section. (I) Sagittal view. bro, birth opening; md, mandible; mo, mouth opening; mx, maxilla; po, paragenital opening; pp, paragenital pouch; pst, prosternum.

The paragenital organ is absent. The relative cuticular thickness could not be assessed with the available µCT data.

#### 3.5.2 *Eurystylops ogloblini* (Figure 6D–I)

The paragenital organ is present. The paragenital opening is sinuate in ventral view, with flat and elongated lateral apical portions. The birth opening has a similar shape as the paragenital opening but is slightly more angular and thus trapezoidal in appearance. Lateral paragenital auricles are absent. The paragenital pouch is weakly developed (12.9% of the cephalothoracic length). The cuticle is markedly thickened along the entire paragenital organ, particularly in the region of the ventral wall. Maximum thickness occurs in the lateral regions of the ventral wall around the lateral opening of the paragenital organ.

#### 3.5.3 *Halictoxenos arnoldi* (Figure 7A–C)

**Figure 7:**
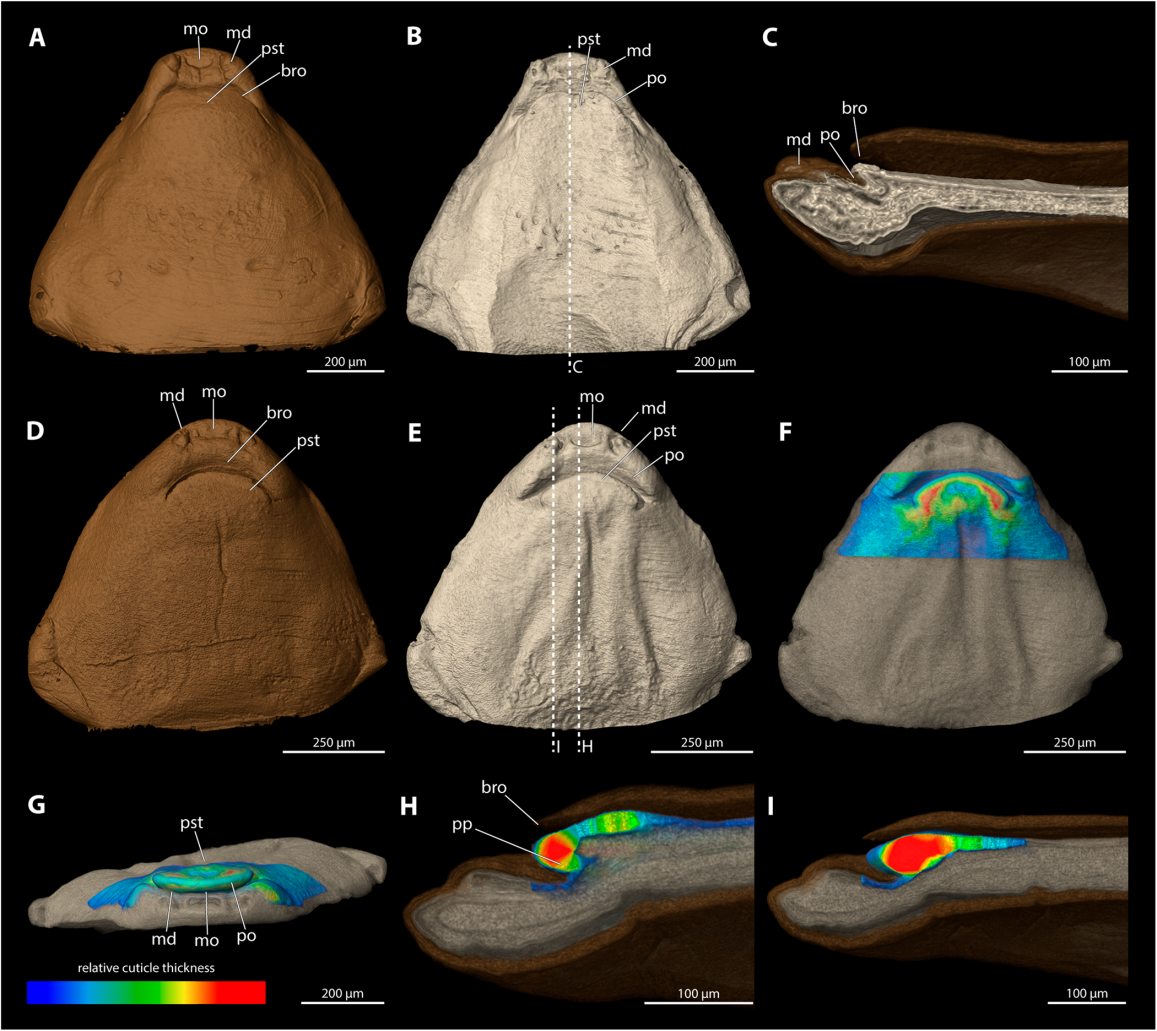
Female cephalothoraces of *Halictoxenos arnoldi* (A–C) and *Halictoxenos simplicis* (D–I), with the ventral side facing upwards. Relative thickness of the cuticle indicated by colour. Blue areas represent thin cuticular regions, while red areas represent thickened areas. The available µCT data did not allow to assess the relative cuticular thickness of *H. arnoldi*. (A, D) Exuvia of the secondary larva. (B, E) Female cephalothorax, exuvia of secondary larva removed. (C) Female cephalothorax (mediosagittal section). (F) Female cephalothorax. (G) Frontal view. (H) Mediosagittal section. (I) Sagittal view. bro, birth opening; md, mandible; mo, mouth opening; po, paragenital opening; pp, paragenital pouch; pst, prosternum.

The paragenital organ is present. The paragenital opening appears as a flattened arch in ventral view. The birth orifice mirrors the shape of the paragenital opening. The lateral auricles are absent. The paragenital pouch is weakly developed and does not extend far into the cephalothorax (3.3% of the cephalothoracic length). The cuticle thickness could not be assessed with the available µCT data.

#### 3.5.4 *Halictoxenos simplicis* (Figure 7D–I)

The paragenital organ is present. The paragenital opening is arched and strongly wide‐gaping. The birth opening above is much narrower but similarly shaped. The lateral auricles are absent, and the pouch is shallow (11.4% of the length of the cephalothorax). The ventral wall of the paragenital organ is strongly thickened, forming a massive, mushroom‐shaped lobe with the anteriormost portion of the brood canal. The thickness of the cuticle in the anterior brood canal decreases rapidly and continuously toward the posterior end. The dorsal wall of the paragenital organ is thin and completely covered by parts of the exuvia of the secondary larva.

#### 3.5.5 *Hylecthrus rubi* (Figure 8A–F)

**Figure 8:**
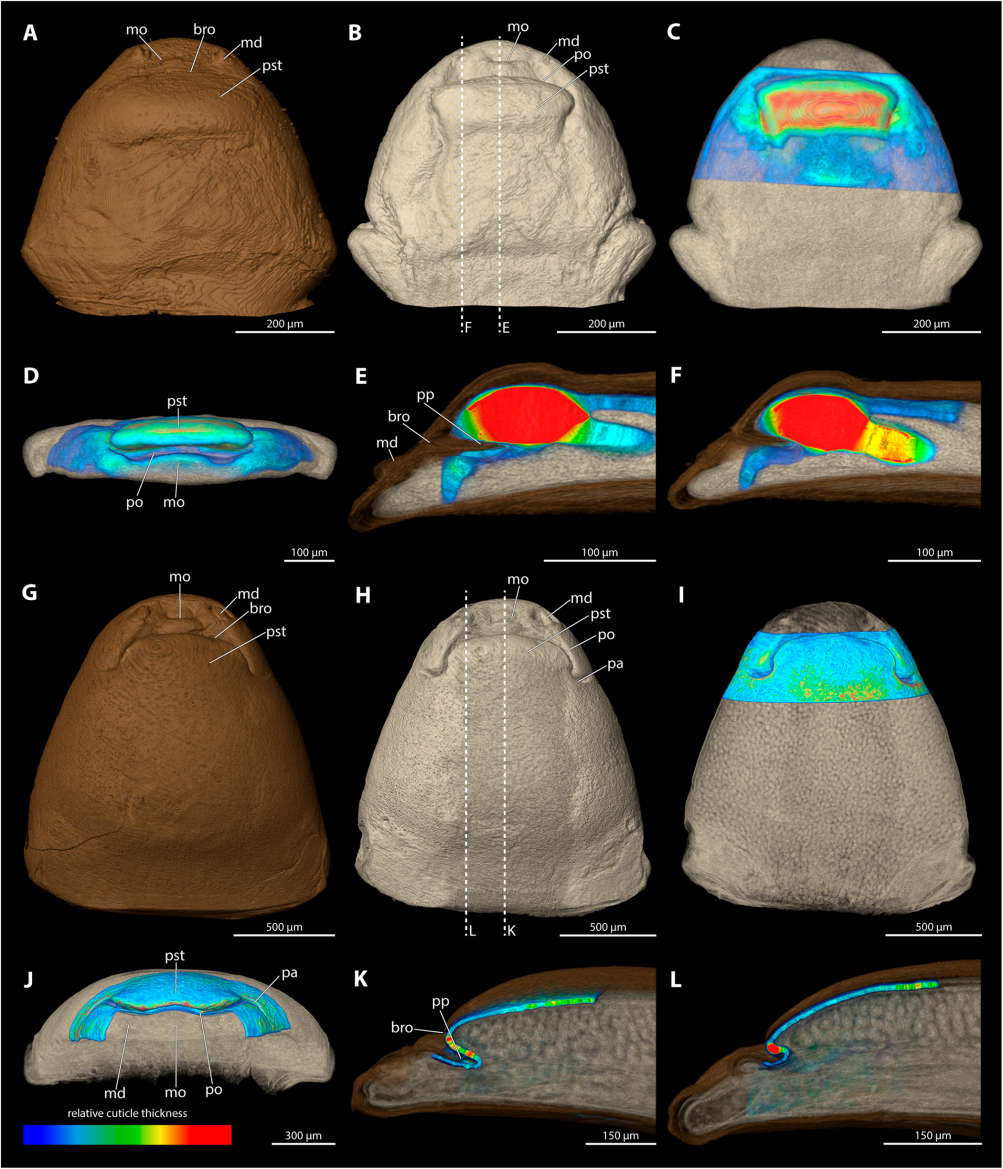
Female cephalothoraces of *Hylecthrus rubi* (A–F) and *Kinzelbachus friesei* (G–L), with the ventral side facing upwards. Relative thickness of the cuticle indicated by colour. Blue areas represent thin cuticular regions, while red areas represent thickened areas. (A, G) Exuvia of the secondary larva. (B, H) Female cephalothorax, exuvia of secondary larva removed. (C, I) Female cephalothorax. (D, J) Frontal view. (E, K) Mediosagittal section. (F, L) Sagittal view. bro, birth opening; md, mandible; mo, mouth opening; pa, paragenital auricles; po, paragenital opening; pp, paragenital pouch; pst, prosternum.

A paragenital organ is present. The opening is slightly curved and bent posterad. The birth opening above it is straight and extends transversely over the opening of the paragenital organ without lateral posterior extensions. The paragenital pouch extends deeply into the cephalothorax (25% of the length of the cephalothorax). The entire ventral wall of the paragenital organ is extremely thick and forms a massive trapezoidal lobe with the anteriormost portion of the brood canal. The thickness of the cuticle in the anterior brood canal decreases abruptly about 100 µm posterior to the paragenital opening. The dorsal wall of the paragenital organ is thin and mostly covered by parts of the exuvia of the secondary larva.

#### 3.5.6 *Kinzelbachus friesei* (Figure 8G–L)

The paragenital organ is present. Its opening is strongly arched with flat lateral apical portions. The pouch of the paragenital organ is shallow and does not extend deeply into the body cavity. The birth opening above it is similar in shape to the paragenital opening. The lateral auricles are weakly developed. The paragenital opening is shallow (6.3% of the length of the cephalothorax). The thickness of the ventral wall of the paragenital organ varies only slightly but more distinctly than the dorsal wall of the paragenital organ and the anterior parts of the brood canal. The dorsal wall of the paragenital organ is largely covered by parts of the exuvia of the secondary larva.

#### 3.5.7 *Stylops hammella* (Figure 9A–F)

**Figure 9:**
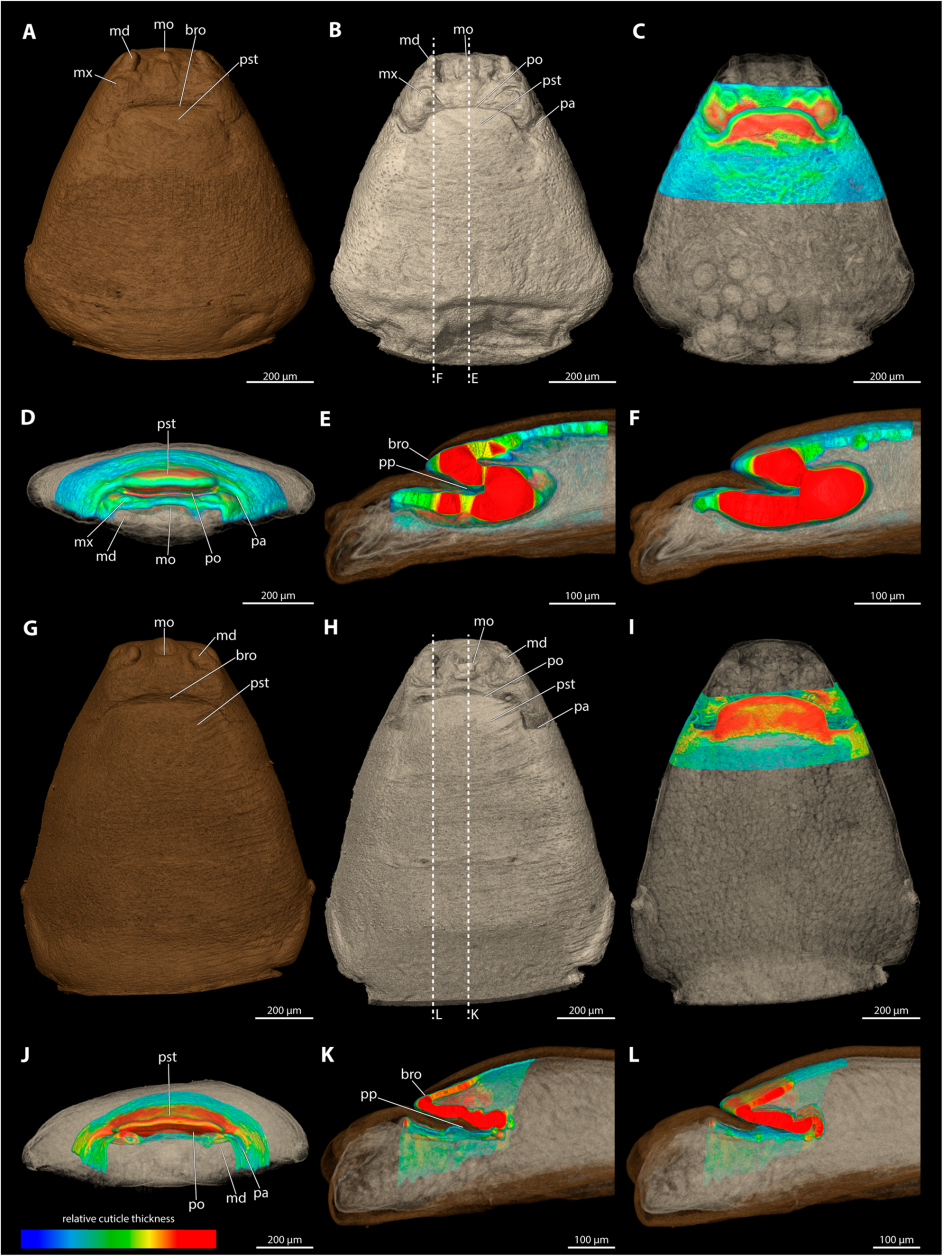
Female cephalothoraces of *Stylops hammella* (A–F) and *Stylops sapmiensis* (G–L), with the ventral side facing upwards. Relative thickness of the cuticle indicated by colour. Blue areas represent thin cuticular regions, while red areas represent thickened areas. (A, G) Exuvia of the secondary larva. (B, H) Female cephalothorax, exuvia of secondary larva removed. (C, I) Female cephalothorax. (D, J) Frontal view. (E, K) Mediosagittal section. (F, L) Sagittal view. bro, birth opening; md, mandible; mo, mouth opening; mx, maxilla; pa, paragenital auricles; po, paragenital opening; pp, paragenital pouch; pst, prosternum.

The paragenital organ is present. The opening is slightly convex with a flattened lateral apical portion. The birth opening above it is similar in shape to the paragenital opening. The lateral auricles are weakly developed and covered by the exuvia of the secondary larva. The paragenital pouch extends deeply into the body cavity (20.6% of cephalothorax length). Both the ventral and dorsal walls of the paragenital organ are extremely thick compared to the surrounding integument. Relative differences between the dorsal wall and the anterior parts of the brood canal are subtle but clearly noticeable. The anterior part of the dorsal wall of the paragenital organ is covered by the exuvia of the secondary larva.

#### 3.5.8 *Stylops sapmiensis* (Figure 9G–L)

The paragenital organ is present. The opening is arched with a flattened lateral apical portion. The birth opening above it has exactly the same shape as the paragenital opening. The lateral auricles are well developed. The paragenital pouch extends deep into the body cavity (16.7% of the length of the cephalothorax). The ventral wall of the paragenital organ is much thicker than the surrounding integument. The relative differences of the thickness of the dorsal wall and the paragenital organ or the anterior areas of the brood canal are pronounced. The wall of the anterior areas of the brood canal is thickened but the cuticle thins out rapidly towards the posterior end. The dorsal wall of the paragenital organ is largely covered by the exuvia of the secondary larva.

#### 3.5.9 *Stylops melittae* (Figure 10A–F)

**Figure 10:**
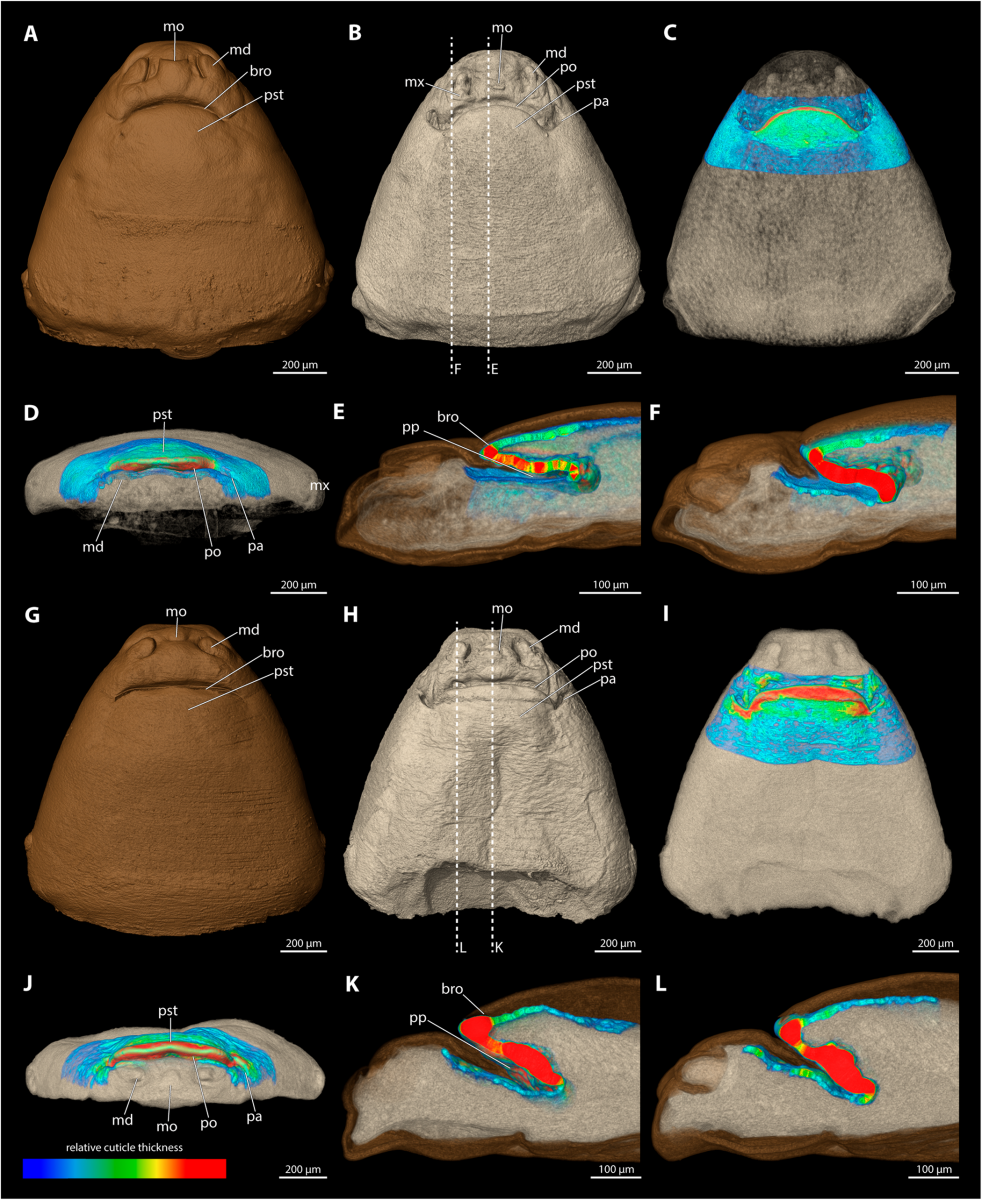
Female cephalothoraces of *Stylops melittae* (A–F) and *Stylops nassonowi* (G–L), with the ventral side facing upwards. Relative thickness of the cuticle indicated by colour. Blue areas represent thin cuticular regions, while red areas represent thickened areas. (A, G) Exuvia of the secondary larva. (B, H) Female cephalothorax, exuvia of secondary larva removed. (C, I) Female cephalothorax. (D, J) Frontal view. (E, K) Mediosagittal section. (F, L) Sagittal view. bro, birth opening; md, mandible; mo, mouth opening; mx, maxilla; pa, paragenital auricles; po, paragenital opening; pp, paragenital pouch; pst, prosternum.

The paragenital organ is present, with an arched opening. The lateral auricles are well developed. The birth opening mirrors the paragenital opening but completely covers the auricles. The paragenital pouch is deep, extending distinctly into the cephalothoracic lumen (21% of the length of the cephalothorax). The ventral wall is much thicker than the surrounding integument; these differences are much more pronounced than in the region of the dorsal wall or the anterior brood canal. Posterior to the anterior brood canal, the thickness decreases rapidly. The dorsal wall of the paragenital organ is largely covered by the exuvia of the secondary larva.

#### 3.5.10 *Stylops nassonowi* (Figure 10G–L)

The paragenital organ is present. The opening is nearly straight, with only a slight curvature. The lateral auricles are well developed. The birth opening is identical in shape to the paragenital opening but completely covers the auricles. The paragenital pouch is prominent and extends well into the cephalothoracic lumen (20% of the length of the cephalothorax). The ventral wall of the paragenital organ is distinctly thicker than its dorsal wall, or the integument of the anterior brood canal. Posterior to the anterior brood canal, the thickness decreases rapidly but the wall does not become as thin as the dorsal cuticle. The dorsal wall is largely covered by the exuvia of the secondary larva.

#### 3.5.11 *Stylops ovinae* (Figure 11A–F)

**Figure 11:**
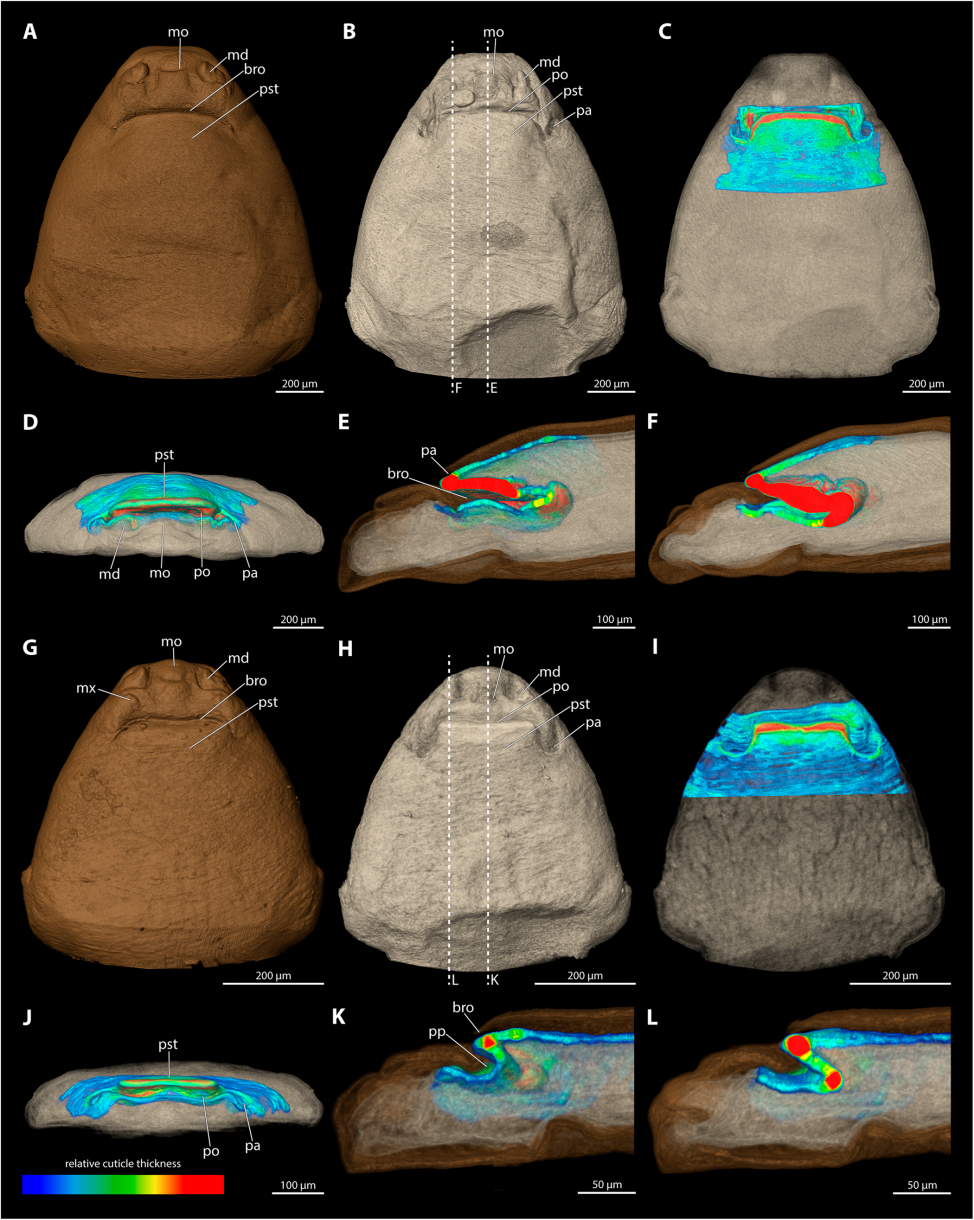
Female cephalothoraces of *Stylops ovinae* (A–F) and *Stylops spretus* (G–L), with the ventral side facing upwards. Relative thickness of the cuticle indicated by colour. Blue areas represent thin cuticular regions, while red areas represent thickened areas. (A, G) Exuvia of the secondary larva. (B, H) Female cephalothorax, exuvia of secondary larva removed. (C, I) Female cephalothorax. (D, J) Frontal view. (E, K) Mediosagittal section. (F, L) Sagittal view. bro, birth opening; md, mandible; mo, mouth opening; mx, maxilla; pa, paragenital auricles; po, paragenital opening; pp, paragenital pouch; pst, prosternum.

The paragenital organ is present. The opening is nearly straight, with only a slight curvature laterally. The lateral auricles are well developed and follow the curvature of the opening. The paragenital pouch extends deeply into the body lumen (20.7% of the length of the cephalothorax). The birth opening is similar to the paragenital opening but entirely covers the auricles. The ventral wall is strongly thickened compared to the dorsal wall and the wall of anterior brood canal. The cuticle is thicker in the sagittal sections of the ventral wall than in its medial sections. Compared to the slightly thickened anterior wall of brood canal, the thickness decreases posteriorly but never matches that of the dorsal paragenital cuticle. The dorsal wall is nearly as thick as the anterior brood canal, and only its anterior region is covered by the exuvia of the secondary larva.

#### 3.5.12 *Stylops spretus* (Figure 11G–L)

The paragenital organ is present. Its opening is slightly concave at the lateral apical portion and trapezoidal in outline. The lateral auricles are well developed. The paragenital pouch is only moderately deep (12.1% of the length of the cephalothorax). The birth opening above it is largely straight, with a slight lateral curvature, and it completely covers the auricles. The ventral wall is thickened, more distinctly sagittally than medially. Compared to the slightly thickened wall of the anterior brood canal, the thickness decreases posteriorly but the ventral wall it is never as thin as the dorsal cuticle. The dorsal wall is as thick as the cuticle of the anterior brood canal and is mostly covered by the exuvia of the secondary larva.

### 3.6 Description of the Intromittent Organs and Possible Correlations With the Paragenital Organ

#### 3.6.1 *Stylops hammella* (Figure 12F)

**Figure 12:**
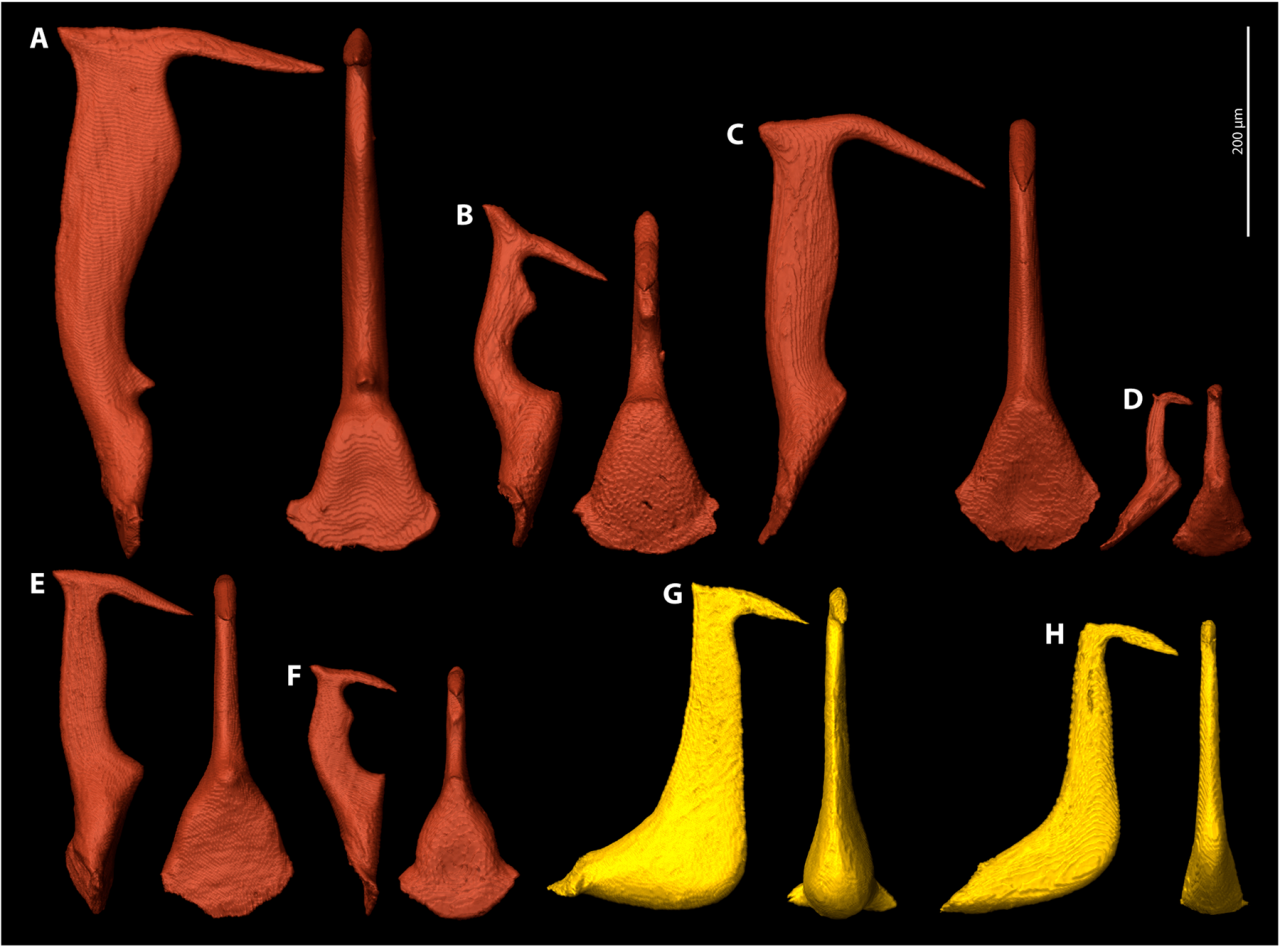
3D reconstructions of penises of Stylopidae (red) and Xenidae (yellow) in lateral view (left) and caudal view (right). (A) *Stylops ovinae*. (B) *Stylops melittae*. (C) *Stylops nassonowi*. (D) *Stylops spretus*. (E) *Stylops sapmiensis*. (F) *Stylops hammella*. (G) *Xenos vesparum*. (H) *Tuberoxenos sphecidarum*. An interactive version of these models is available in the HTML version of this article online and on Sketchfab at: https://sketchfab.com/entomology_uni_jena/collections/strepsiptera-penises-79603a06f3c74a2aacf76d98e875202e.

The penis of *S. hammella* is hook‐shaped and dorsoventrally compressed. Its base is broad ventrally, narrowing toward the scapus aedoeagi. The scapus aedoeagi bears a prominent bulge on its frontal ridge, and a well‐developed dorsal spine is present.

#### 3.6.2 *Stylops sapmiensis* (Figure 12E)

The penis is hook‐shaped with a well‐developed dorsal spine. The scapus aedoeagi is attached at a right angle to the penis base. A bulge on the scapus aedoeagi is only slightly developed.

#### 3.6.3 *Stylops melittae* (Figure 12B)

The penis of *S. melittae* is hook‐shaped, and the scapus aedoeagi is oriented at a 90° angle to the penis base (Figure 12B). A bulge is present on the frontal side of the scapus aedoeagi. The penis base is broad and tapers towards the scapus aedoeagi. It lacks a frontal spine but bears a prominent dorsal spine.

#### 3.6.4 *Stylops nassonowi* (Figure 12C)

The penis is slender and bears a well‐developed dorsal spine. The penis base is broadened and narrows towards the scapus aedoeagi. A conspicuous feature of the acumen is that it bends ventrad.

#### 3.6.5 *Stylops ovinae* (Figure 12A)

The penis of *S. ovinae* is hook‐shaped (Figure 12A). The base is broadened in frontal view. Two prominent spines are present dorsally and frontally. The scapus aedoeagi is conspicuously bloated.

#### 3.6.6 *Stylops spretus* (Figure 12D)

The minute penis of *S. spretus* is hook‐shaped, with a tiny dorsal spine. Its base forms an obtuse angle with the scapus aedoeagi and is broad ventrally and narrowing toward the scapus aedoeagi. The acumen is conspicuously short.

#### 3.6.7 *Paraxenos erberi* (Figure 12H)

The penis is hook‐shaped and lacks any spines. The base is slender and narrows gradually toward the acumen.

#### 3.6.8 *Xenos vesparum* (Figure 12G)

The penis of *X. vesparum* is hook‐shaped. It has a moderately prominent dorsal spine. The base is bulbous and massive.

**Figure 13:**
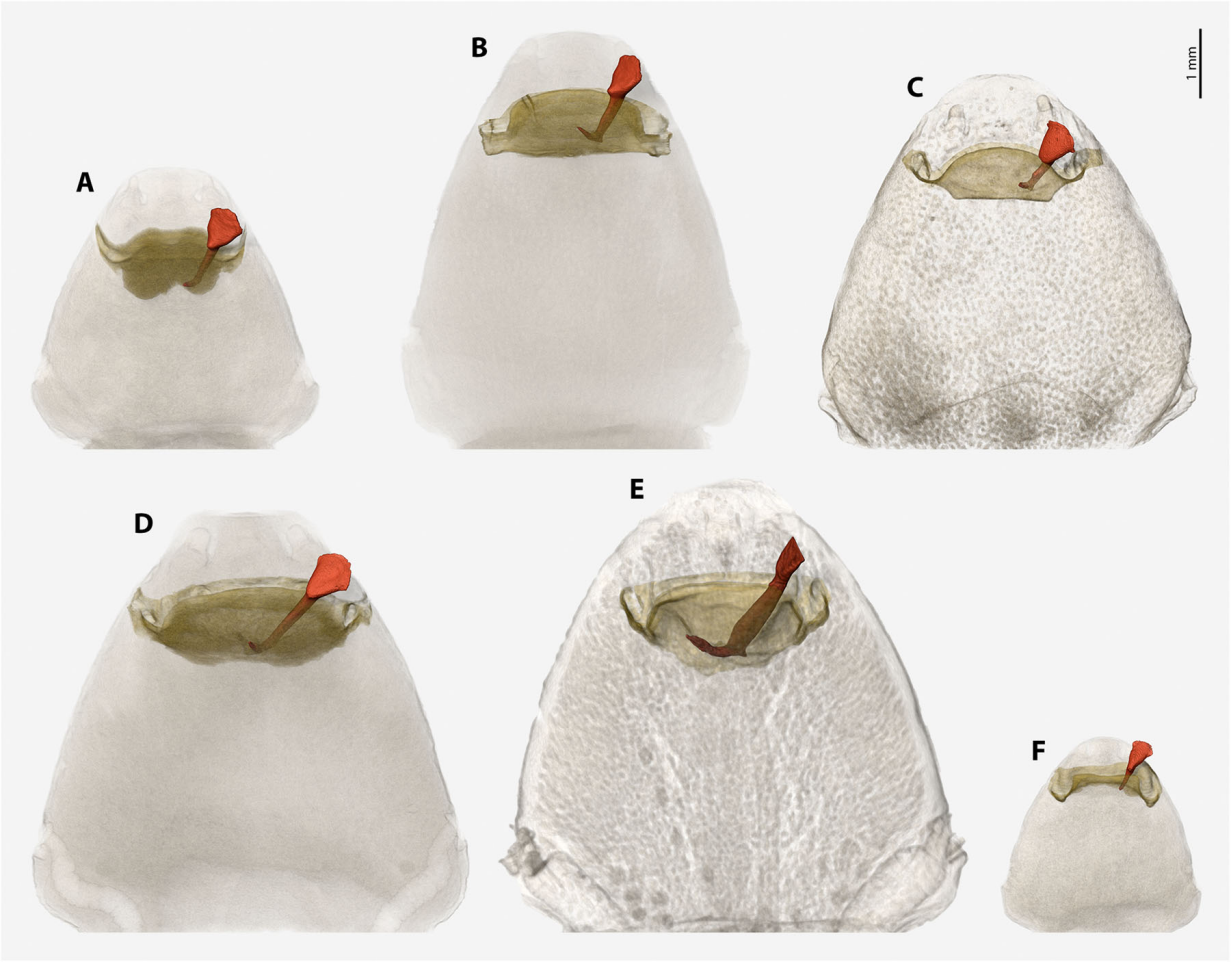
Pairs of female cephalothoraces (outer cuticle of the cephalothorax removed) and penises. (A) *Stylops hammella*. (B) *Stylops sapmiensis*. (C) *Stylops melittae*. (D) *Stylops nassonowi*. (E) *Stylops ovinae*. (F) *Stylops spretus*. Paragenital organs highlighted in ochre, penises in red and orange. *S. ovinae* was scanned in copula (Peinert et al. 2016). The penises of *S. hammella*, *S. sapmiensis*, *S. melittae*, *S. nassonowi*, and *S. spretus* were virtually inserted into the corresponding female paragenital organs of conspecifics to assess the fit of these genital structures. DP, depth of paragenital organ; LA, length of the acumen; PL, length of the penis.

**Figure 14:**
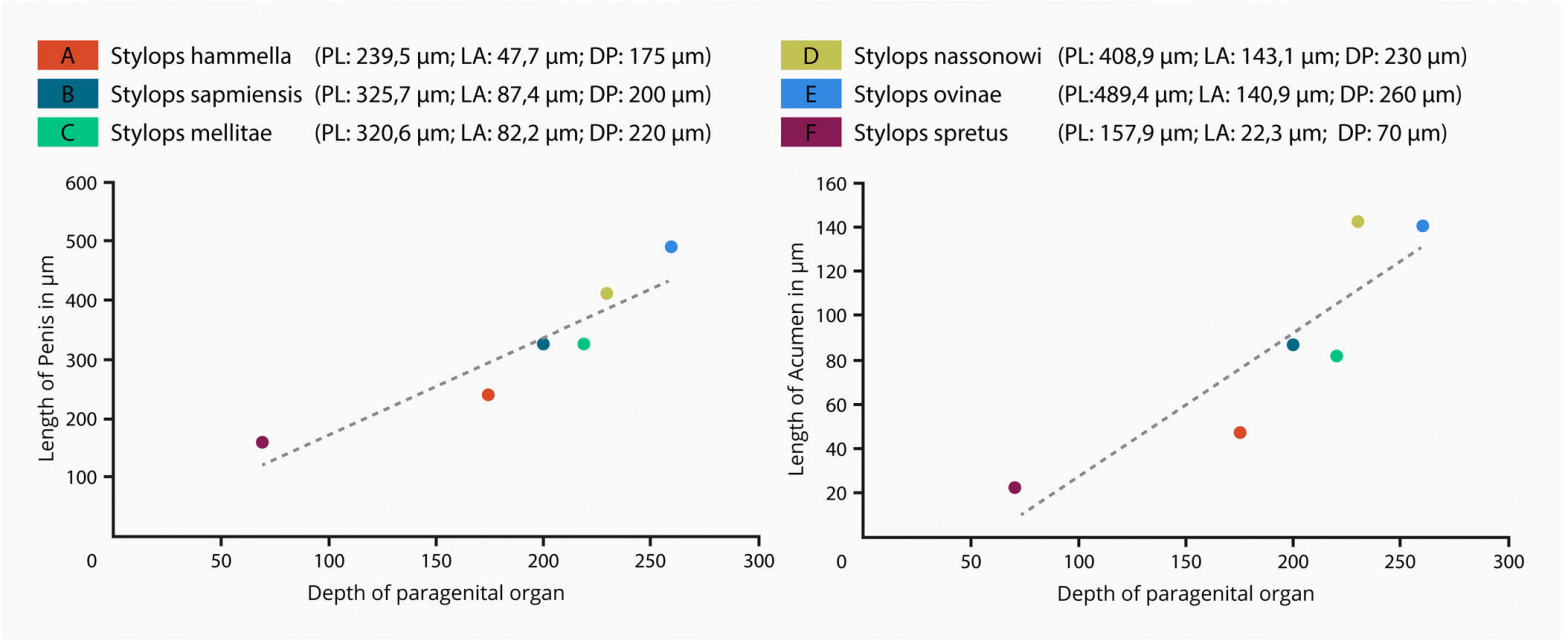
Correlation of the depth of the paragenital organ with the length of the penis (left) and the length of the acumen (right). DP, depth of paragenital organ; LA, length of the acumen; PL, length of the penis.

The virtual insertion of the penis of the studied *Stylops* species demonstrated a perfect fit between length of the penis (PL) and depth of the paragenital organ of a female of the same species (DP) (Figure 13A–F). This is underlined by measurements of the depth of the paragenital organ and length of the penis which revealed a positive correlation in size (Figure 14), that is, greater depth of the paragenital organ corresponds with a longer penis. A positive correlation was also found between the length of the acumen (LA) (e.g. Figure 12A) and the depth of the paragenital organ (DP) (Figure 14).

## 4 Discussion

Based on our results, we define several structures of the paragenital organ—namely the paragenital opening, the paragenital pouch, and the paragenital auricles. Their significance and interpretation for the phylogeny and evolution of Strepsiptera, and more specifically Stylopidae, are discussed in subsequent paragraphs. Furthermore, we characterise the lateral processes of the secondary larval exuvia that extend into the paragenital organ apparently to interact with it.

### 4.1 Function and Interpretation of the Exuvial Lateral Processes

The lateral processes of the secondary larval exuvia, which were described by Kinzelbach (1971) as elements of the tentorium, are related to the paragenital organ. As these structures are not located in the head or cephalic region, we reject the homology statement of Kinzelbach (1971) and suggest a functional relationship between these de novo formed structures and the paragenital organ.

Females of Strepsiptera, with the partial exception of those in the family Mengenillidae, remain within their larval exuvia throughout their adult life. Such long‐term use or re‐use of the larval exoskeleton after moulting is rare but not restricted to Strepsiptera. For example, some lacewing larvae (Neuroptera: Chrysopidae) carry debris for camouflage, including parts of their larval skin (Tauber et al. 2014); turtle beetle larvae (Coleoptera: Chrysomelidae: Cassidinae) use their exuvia to shield their abdomen (Olmstead 1994; Ramos et al. 2019); and certain caddisflies (Trichoptera) that remain in their pupal case as adults employ parts of the pupal integument to form a tendon‐like structure connecting their claws to those of the pupa (e.g., Betten 1934; Hinton 1946; Friedrich and Kubiak 2018). While the functional link between adult caddisflies and their exuvia is remarkable, the lifelong functional unit formed by an adult female Strepsiptera and the secondary‑larval exuvia remains unique.

Some morphological peculiarities arising from a lifelong functional unit formed by adult female Strepsiptera with their secondary larval exuvia have been described in detail (e.g., Pohl and Beutel 2005; Pohl and Beutel 2008; Löwe and Beutel 2016; Richter et al. 2017). These include the brood canal of Stylopidia and the pores on the ventral side of the larval exuvia, which apparently serve as openings for Nassonov's glands (Löwe and Beutel 2016). The function of another structural element, the processes laterad the paragenital organ in the second larval exuvia, remains to be investigated in more depth. These lateral processes occur only in females of Stylopidae with a paragenital organ. We found that the length of these processes increases with the depth of the paragenital organ. For example, females of *Eurystylops* have only a shallow paragenital pouch and weakly developed lateral exuvial processes. In contrast, in *Stylops* species with much more pronounced exuvial processes the paragenital pouch extends deeply into the cephalothoracic lumen. This suggests that the lateral projections stabilise the pouch and keep it open for penetration. If the dorsal and ventral walls of the paragenital organ would collapse due lack of mechanical support, penetration by the penis would be impeded, thus reducing the chance of successful fertilisation.

### 4.2 Phylogenetic Distribution of the Paragenital Organ

As noted by several authors (Lauterbach 1954; Kinzelbach 1971; Kinzelbach 1978; Löwe and Beutel 2016; Jandausch et al. 2022; Jandausch et al. 2023), a paragenital organ is present in all species of *Stylops*. The organ varies in length, depth of the pouch, and shape of the opening. Distinct auricles at the lateral ends of the paragenital opening are characteristic for *Stylops* compared to species of other genera of Stylopidae.

We also detected a paragenital organ in some species of the genera *Eurystylops*, *Halictoxenos, Hylecthrus*, and *Kinzelbachus* of Stylopidae. In these genera, the organ differs from that of *Stylops* in two ways: (I) it extends less deeply into the female cephalothorax, (II) lateral auricles are entirely absent. An exception is *Crawfordia*, whose species lack a paragenital organ altogether.

Considering our results in the context of recent phylogenetic studies (Jůzová et al. 2015; Pohl et al. 2021; Benda et al. 2022; Jandausch et al. 2023), we interpret the paragenital organ as a potential autapomorphy of Stylopidae. *Kinzelbachus*, in which we identified a paragenital organ, was considered the sister group of all other Stylopidae (Jůzová et al. 2015), suggesting that this feature belongs to the groundplan of the family (new phylogenomic analyses place *Eurystylops* as sister to *Stylops* (Lähteenaro et al. 2024)). *Crawfordia*, the only genus of Stylopidae without a paragenital organ, is nested within the family. We therefore suggest that this structure is secondarily missing in females of this genus. However, the reasons for this reduction remain unclear. Our data suggest that the lateral auricle of the paragenital organ is an autapomorphy of Stylopidae. Morphological studies of females in other families showed that comparable structures do not occur outside of Stylopidae (e.g., Kirkpatrick 1937; Silvestri 1943; Baumert 1958; Kinzelbach 1971; Pohl and Beutel 2005; Pohl and Beutel 2008; Pohl et al. 2012; Löwe and Beutel 2016; Tröger et al. 2023).

### 4.3 Significance of the Paragenital Organ for Copulation

Traumatic insemination is costly for mated females (Morrow and Arnqvist 2003; Lange et al. 2013; Tatarnic et al. 2014; Michels et al. 2015; Reinhardt et al. 2015). Paragenital organs occur in many species of Cimicoidea (Heteroptera) and are clearly associated with traumatic insemination (Tatarnic et al. 2014; Reinhardt et al. 2015; Jung et al. 2023). Traumatic insemination is the general copulatory mode in Strepsiptera, but a paragenital organ is restricted to Stylopidae. Therefore, it is not, or at least not exclusively, an adaptation that reduces costs for females. Instead, simple cuticular thickening likely represents a cost‐reducing adaptation. This is consistent with microindentation experiments carried out with *S. ovinae* and *X. vesparum*, where Jandausch et al. (2022) showed that the females can improve tolerance of the damage of traumatic insemination by thickening their integument. As shown in other studies (Richter et al. 2017; Jandausch et al. 2022; Jandausch et al. 2023), we observed thickening of the integument in the anterior brood canal of *X. vesparum* (Xenidae). However, similar thickening at penetration sites has also been documented in Elenchidae (Baumert 1958; Jandausch et al. 2023) and Halictophagidae (Jandausch et al. 2023), indicating that this feature is functionally linked to traumatic insemination across Strepsiptera.

The genus *Triozocera* deserves special attention, as Corioxenidae lack a birth opening, a plesiomorphic groundplan feature of Stylopidia. In Corioxenidae, the penis pierces the sclerotised exuvia of the female secondary larva before penetrating the enclosed female. In other families with a birth opening, i.e., all groups of Stylopiformia (e.g., Xenidae or Stylopidae), the penis also pierces the secondary‐larval exuvia, but at regions where the cuticle is very thin (Löwe and Beutel 2016; Peinert et al. 2016; Richter et al. 2017; Jandausch et al. 2023). Therefore, we expected that the larval exuvia of Corioxenidae is particularly thin at typical penetration sites, namely the oral region and the membranous pleural areas of the anterior cephalothorax. However, interestingly, thin integument is found only in the mouth region, but not in the pleural areas. We propose two alternative explanations for this pattern: 1) mating in *Triozocera* occurs exclusively in the oral region (Kirkpatrick 1937; Pohl and Beutel 2008), or 2) the membranous pleural areas differ in material composition rather than thickness. Variable material composition is known in the bed bug, *Cimex lectularius* (Michels et al. 2015), whose spermalege integument is rich in resilin. Although further studies across more Strepsiptera families and genera are needed to test these hypotheses, we suggest that thickening of the integument, especially of resilin‐rich cuticle, is the primary cost‐reducing adaptation to traumatic insemination in Strepsiptera. Any thickening of the ventral wall of the paragenital organ may simply reflect its role as a penetration site.

### 4.4 Interaction of the Paragenital Organ and the Penis

The paragenital organ of the Stylopidae plays a crucial role as a penetration site during copulation, as convincingly demonstrated by Peinert et al. (2016), who also suggested that it reduces the cost of injuries from multiple matings. However, Jandausch et al. (2022) showed that *S. ovinae* females attract heterospecific males via a pheromone signal (Cvačka et al. 2012; Tolasch et al. 2012) but cannot mate with them. Consequently, Jandausch et al. (2022) hypothesised that the paragenital organ prevents heterospecific mating. Our data provide further support for this interpretation.

The variability of the paragenital organs and the variation in penis shape among different *Stylops* species (paragenital organs: Figures 7, 8, 9; penises: Figure 12) suggest a prezygotic mating barrier. This barrier may result from a mechanical interaction between the paragenital pouch and the penis (lock and key). The length of the acumen (Figure 12A) and the total penis length, penetrating the thickened integument of the paragenital organ, may contribute to this barrier (Figure 13G). It is conceivable that these factors have a cumulative effect. Whether similar mechanical barriers prevent heterospecific mating in other Strepsiptera genera should be addressed in future studies.

## 5 Conclusion

We propose that the presence of a female paragenital organ is an autapomorphy of Stylopidae. This structural complex is absent in the species of Xenidae we studied—the sister group of Stylopidae—as well as in other Stylopidia families. Compared to the surrounding cuticle, the paragenital organ is characterised by pronounced structural modifications, notably a variously deep pouch and distinct thickening of the cuticle. We further showed that in many species with a paragenital organ, the exuvia of the secondary larval develops mechanically rigid processes with a novel function: stabilising the paragenital pouch. Paragenital auricles related with this function evolved within Stylopidae, and were strengthened in *Stylops*, the latter condition likely an autapomorphy of this genus. Mechanical reinforcement of the paragenital organ acilitates intromission of the male penis and thus increases the chance of successful fertilization. Finally, we suggest a prezygotic mating barrier arising from the variability of paragenital organs and corresponding differences in penis shape among *Stylops* species.

## Author Contributions

**Kenny Jandausch:** methodology, investigation, writing – original draft, visualization, validation, writing – review and editing, formal analysis, resources, data curation. **Jakub Straka:** writing – review and editing, resources. **Thomas van de Kamp:** writing – review and editing, investigation, methodology, resources. **Heiko Stark:** writing – review and editing, investigation, methodology, software. **Rolf G. Beutel:** writing – review and editing, conceptualization, funding acquisition, supervision, resources. **Oliver Niehuis:** writing – review and editing, supervision, resources, funding acquisition, conceptualization, writing – original draft, project administration, methodology, validation, investigation. **Hans Pohl:** writing – review and editing, writing – original draft, conceptualization, investigation, funding acquisition, methodology, validation, project administration, supervision, data curation, resources.

## Conflicts of Interest

The authors declare no conflicts of interest.

## Peer Review

The peer review history for this article is available at https://www.webofscience.com/api/gateway/wos/peer-review/10.1002/jmor.70088.

## Data Availability

The data that support the findings of this study are available on request from the corresponding author.
